# CRISPR screens identify PRMT7 as a therapeutic target to enhance T cell-mediated killing in breast cancer

**DOI:** 10.1038/s41523-025-00888-8

**Published:** 2026-01-21

**Authors:** Wei Shi, Yi Luo, Yizhuo Wang, Jacqueline M. Burrows, Debra Black, Andrew Civitarese, Laura Perlaza-Jimenez, Ping Zhang, Murray Manning, Natasha Tuano, Miguel E. Rentería, Christos Xiao, Siok-Keen Tey, Joseph Rosenbluh, Corey Smith, Georgia Chenevix-Trench, Jonathan Beesley

**Affiliations:** 1 Cancer Research Program, QIMR Berghofer, Brisbane, QLD Australia; 2 Faculty of Medicine, University of Queensland, Brisbane, QLD Australia; 3 Infection and Immunology Program, QIMR Berghofer, Brisbane, QLD Australia; 4 Cancer Research Program and Department of Biochemistry and Molecular Biology, Biomedicine Discovery Institute, Monash University, Clayton, VIC Australia; 5 Monash Functional Genomics Platform, Monash University, Clayton, VIC Australia; 6 Mental Health and Neuroscience Program, QIMR Berghofer, Brisbane, QLD Australia

**Keywords:** Breast cancer, Genome-wide association studies, Functional genomics, Immunosurveillance

## Abstract

Genome-wide association studies (GWAS) have identified more than 220 loci associated with breast cancer susceptibility, yet identifying effector genes, their modes of action and prioritising therapeutic targets remains a significant challenge. To address this, we performed pooled CRISPR knockout and inhibition screens to identify genes at risk loci that influence cytotoxic T lymphocyte (CTL) killing of MCF7 breast cancer cells in co-culture. These screens uncovered 33 candidate modulating genes, of which we validated six by single gene editing in two cell lines. Deletion of *IRF1*, *ATF7IP*, and *CASP8* conferred resistance to CTL killing, while disruption of *CFLAR*, *CREBBP* and *PRMT7* enhanced sensitivity. Analysis of clinical data showed that *PRMT7* expression is negatively correlated with CD8+ infiltration and survival in breast cancer patient cohorts. Pharmacological inhibition of PRMT7 sensitized breast cells to CTL killing in vitro, and *Prmt7*-deficient tumors exhibited reduced growth and increased CD8+ T cell infiltration in immunocompetent mice. Enhanced Prmt7-dependent tumor growth was not observed in immunodeficient mice, implicating Prmt7 in immune evasion. This study underscores the utility of CRISPR screens for high-throughput functional follow-up of GWAS findings and identifies PRMT7 inhibition as a promising therapeutic strategy.

## Introduction

Genome-wide association studies (GWAS) have identified over 220 loci associated with breast cancer risk^1,2^. Genetic fine-mapping and computational analyses have predicted more than 1200 candidate target genes, including ~200 with high confidence^3^. Pathway analysis of these genes showed that immune system processes, DNA integrity checkpoint and apoptosis were among the top-enriched pathways for both estrogen receptor (ER)-positive and ER-negative subtypes^3^. We have previously performed high-throughput custom CRISPR screens to identify target genes at risk loci that drive cancer hallmarks including modulators of proliferation, tumorigenicity and DNA damage repair^4^. We now extend these screens to identify candidate breast cancer GWAS target genes involved in T cell-mediated immunosurveillance.

Cancer immunoediting consists of three phases: elimination (also known as cancer immunosurveillance), equilibrium, and escape, during which the immune system not only protects against cancer development but also shapes the character of developing tumors^5–9^. Cancer immunosurveillance recognizes neo-antigens on transformed cells and eliminates them before tumors are detectable. This process, involving components of the innate and adaptive immune systems, is essential in early anti-tumor responses to induce cell death and increase tumor immunogenicity^5,6,8^. Cytotoxic T lymphocytes (CTL) are capable of antigen-directed target cell killing and are key mediators of immunosurveillance^10^. While much focus has been placed on understanding the immune escape phase in the context of breast cancer treatment, it is likely that the elimination phase involves cancer risk genes, such as those identified in GWAS, acting in early phases of tumorigenesis^11^.

There have been few studies to date examining immune functions in women genetically predisposed to breast cancer. A recent single-cell transcriptomic analysis of risk-reduction mammoplasties showed that immune cells from *BRCA1/2* carriers had a distinct gene expression signature indicative of potential immune exhaustion, suggesting that immune escape mechanisms could manifest in non-cancerous tissues during very early stages of tumor initiation^12^. In addition, a recent study showed that the anti-tumor response becomes less active during the transition from ductal carcinoma in situ (DCIS) to invasive ductal breast cancer (IDC), with fewer activated granzyme B-expressing CD8+ T cells in IDC than DCIS^13^. The authors reasoned that in DCIS, with the help of a mostly intact physical barrier, the immune cells are able to eliminate the relatively few cancer cells that are contained. Therefore, the elimination process is critical to foil the DCIS-IDC transition, with clinically detected IDC representing a failure of elimination. Furthermore, several strains of immunodeficient mice develop spontaneous mammary carcinomas, demonstrating the importance of elimination^5,14^.

We hypothesized that breast cancer risk genes identified through GWAS may modulate the sensitivity of tumor cells to immune killing. To test this, we performed targeted CRISPR screens in breast cancer cells co-cultured with CTLs, uncovering candidate risk genes with sensitizer or resistor activity, including pharmacologically tractable targets such as protein arginine methyltransferase 7 (*PRMT7*). PRMT7 modifies histone and non-histone substrates to regulate signaling, transcription, and protein interactions, and has been implicated in cellular differentiation, proliferation, stress responses, and breast cancer metastasis^15,16^. Here, we identify PRMT7 as a key intrinsic mediator of breast tumor cell evasion from CTL attack and highlight its potential as a novel target for immune modulation in breast cancer.

## Results

### Selection of genes for the targeted library

**Fig. 1:**
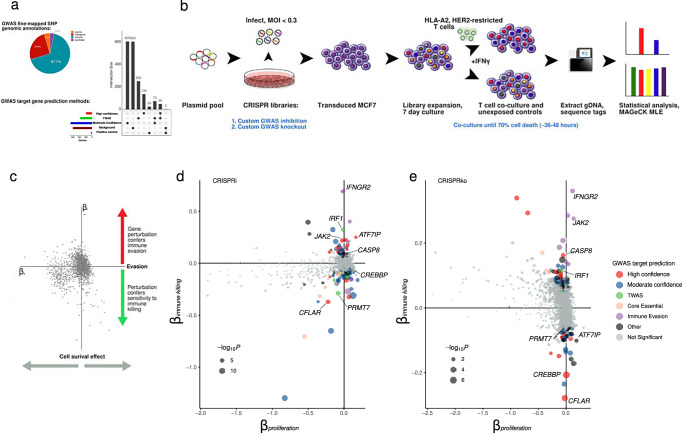
Gene selection, experimental design and workflow to identify candidate breast cancer risk genes which affect cytotoxic T cell-mediated breast tumor cell killing. **a** Genes from breast cancer GWAS and TWAS were selected from previous studies. **b** MCF7 cells were infected with custom GWAS knockout and inhibition libraries before co-culture with T cells. Genomic DNA was extracted from surviving cells and the difference in guide abundance in the unexposed and co-cultured groups analyzed with MAGeCK**. c** Gene-level effects for cell survival (*y*-axis) and T cell exposure (*x*-axis), showing depletion of core essential genes (*β*_proliferation_ < 0) over the course of the experiment. Cytotoxic T cell evasion resistor genes are characterized by *β*_immune killing_ < 0 and sensitizers by *β*_immune killing_ > 0 in CRISPRi (**d**) and CRISPRko (**e**). Genes selected for validation are labelled and are coloured according to gene category (high confidence INQUISIT red, moderate confidence INQUISIT blue, TWAS green, immune evasion positive controls purple).

To build the custom CRISPR libraries, we selected the target genes at 205 fine-mapped breast cancer risk GWAS signals predicted using the INQUISIT pipeline^3^, genes from Transcriptome-wide Association Studies (TWAS) and expression quantitative trait loci studies of breast cancer risk^4^ (Fig. 1a, and Supplementary Data 1). We designed five sgRNAs for each gene, and included 1000 negative control sgRNAs targeting the *AAVS1* region, 1000 sgRNAs targeting 193 core essential genes, and 26 positive control genes known to confer resistance to T cell–mediated killing (Supplementary Data 2).

### Optimization of the CTL assay

In order to identify appropriate target cell lines for the screen, we compared the cytolysis efficacy of HER2-restricted T cells against four HLA-A2 positive breast cell lines, MCF7, SKBR3, MDA-MB-231 and B80T5 (Fig. S1a). MCF7 was the cancer cell line most sensitive to HER2-restricted T cells with a 20 h delay in onset, followed by an almost linear killing curve, achieving 90% lysis after a 52 h co-culture period. In comparison, SKBR3 and MDA-MB-231 were more resistant to HER2-restricted T cells with the cytolysis rate plateauing at about 30% (Fig. S1a). Previous studies have shown an inverse correlation between HER2 expression and MHC-I levels in breast tumor tissues, MDA-MB-231 cells^17^ and other cancer cells^18,19^. We hypothesized that insufficient antigen presentation on the cell surface of breast cancer cells may impede CTL killing. To test this, we compared the expression levels of HER2 and antigen presentation molecules HLA-ABC on the surface of MCF7, B80T5, MDA-MB-231 and SKBR3 cells. Indeed, SKBR3 cells had the highest expression of HER2 both at basal state and after IFNγ stimulation (Fig. S1b). Although IFNγ further increased HLA expression in all four cell types, SKBR3 expressed low levels of HLA relative to MCF7 (Fig. S1b). While MCF7 is not classified as an HER2-amplified cell line, it may have elevated antigen presentation via MHC-I, leading to a more efficient HER2-specific CTL activation. Therefore, we selected the HER2^moderate^ cell line, MCF7, to study the activation of CTL killing to avoid the confounding effect of HER2-mediated downregulation of tumor antigen presentation in HER2^high^ cells.

Increased HLA class I expression is associated with increased efficiency of antigen processing and presentation^20–25^. We next tested the concentrations of IFNγ for optimal tumor antigen presentation. Consistent with previous studies^26^, we found that MCF7 treated with 10 ng/ml of IFNγ for 24 h robustly upregulated HLA-ABC expression (Fig. S1c). We also compared T cell killing efficiency by altering E:T ratios from 0.25 to 8, and showed that a ratio of 1:1 exerted intermediate immune pressure (Fig. S1d), suitable for the phenotypic screen endpoint. By comparing T cell killing efficiency of HER2-restricted T cells versus T blasts, we showed the specificity of our assay (Fig. S1e). Following this optimization, we used the following co-culture conditions for the screen: HER2-restricted T cells and target MCF7 cells at a 1:1 ratio, with target cell IFNγ pre-treatment, and an endpoint of 70% cytolysis in the parallel xCELLigence assay.

### Identification of candidate breast cancer risk genes which regulate sensitivity to T cell cytotoxicity

To gain a comprehensive view of breast cancer GWAS target genes associated with immune evasion, we performed functional CRISPR screens, using custom CRISPRi and CRISPRko libraries (Fig. 1b). Following independent library infections in triplicate cultures, cells were grown for 7 days to deplete cells with essential gene perturbations. MCF7 cells were then co-cultured with HER2-restricted T cells, and when 70% cytolysis was observed, cells were harvested and genomic DNA used for quantification of sgRNA abundance. Sequencing analysis revealed sgRNA library mapping rates >90% and Gini index <0.1 for all sample libraries. Consistent read distributions indicated that guides were adequately represented at all stages of the workflow. To identify genes that mediate response to T cells, we compared sgRNA abundance in co-cultured cells and unexposed controls using the MAGeCK pipeline^27^ (Fig. 1c, d). We further assessed screen quality by examining the levels of sgRNAs targeting 193 core essential genes. Compared to early experimental timepoints (day 3), the log read count of essential genes relative to non-essential genes was substantially left-shifted in later time points (up to day 11), demonstrating the reliability of inhibition and knockout screens (Fig. S2a, c). We further distinguished between candidate CTL evasion effects and genes with functions that promote survival in MCF7. We retrieved fitness genes from the Cancer Dependency Map Project^28^ and designated MCF7 essential genes as *Chronos score* < −1 (892 genes, of which 226 were in the screen library). We found that mean sgRNA abundances for the genes known to contribute to MCF7 survival in culture were again left-shifted relative to non-essential screen genes (Fig. S2b, d).

We used the maximum likelihood estimation (MLE) approach to model experimental conditions and compute an effect score (‘β‘) which reflects the extent of selection (Supplementary Data 3). To account for fitness effects, we modeled a variable to account for general effects on cellular fitness in culture in our screen data (Fig. S3). We set an adjusted significance threshold of 0.2 to identify candidate breast cancer risk genes that act as “sensitizers” to T cell killing (*β* > 0, relative sgRNA enrichment in surviving cells), and “resistors” that enable MCF7 cells to evade T cell cytotoxicity (*β* < 0, sgRNA depletion). As expected, negative controls had no effect on cytotoxicity. We observed positive correlation between the CRISPRi and CRISPRko screens (*ρ* = 0.25; *P* < 2.22 × 10^−16^), indicating that gene perturbation using different modalities resulted in concordant effects (Fig. S4). We identified 88 genes which were significantly enriched or depleted upon T cell-mediated tumor cell killing (FDR < 0.2, Supplementary Table 3). Positive control genes were detected in the inhibition (*CD58, IFNGR1, IFNGR2, JAK1, JAK2, NLRC5* and *TAP1*) and knockout screens (*CD58, HLA-A, IFNGR1, IFNGR2, JAK1, JAK2*, and *TAP1*), and were associated with positive *β* values (Supplementary Table 3), indicating that perturbation induced resistance to immune killing. Eleven genes were detected in both screens.

To differentiate between genes essential for cell survival and probable CTL genes, we examined sgRNA abundances across all treatment conditions compared to baseline. We found 9 and 26 genes (FDR < 0.05) with negative *β* values for the fitness variable (indicating depletion over time in culture) in the knockout and inhibition screens, respectively, which are likely to be genes depleted due to effects on cell survival rather than CTL evasion. These genes include GWAS targets *CCND1, ESR1, GATA3* and *EWSR1*. As expected, essential genes were enriched in core pathways such as DNA repair and proteasome degradation pathways. Using data from the DepMap Project, 31 genes known to affect MCF7 fitness were not considered as putative CTL evasion genes. The final filtered list of candidate GWAS target genes with an effect on T cell killing comprised 20 resistors and 13 sensitizers (Table 1). Of these, 25 were predicted to be target genes at GWAS signals by INQUISIT, and 8 by TWAS. Using the library content as background, analysis using the Enchichr tool showed the 33 CTL genes were enriched for mSigDB Hallmark pathway terms including Apoptosis and TNFα signaling (Adjusted *P* values < 0.05). Of note, several genes are designated as tractable targets and a subset have known small molecule inhibitors (e.g., *CASP8*, *CREBBP*) or antibody antagonists (*IL6ST*). The genes we identified as resistors may be suitable for pharmacological inhibition and represent novel cancer immunotherapy targets.

**Table 1:** Summary of CTL evasion genes

Gene ^a^	Gene selection criteria	Screen modality	CTL evasion effect	Breast cancer risk association ^b^	Tractability ^c^	Known inhibitors ^d^
ATF7IP*	High confidence (INQUISIT 1)	CRISPRi	sensitizer	Decreased expression^4^		
CASP8*	High confidence (INQUISIT 1)	CRISPRko	sensitizer	Decreased expression^34^	Small molecule	NIVOCASAN, EMRICASAN
CFLAR*	High confidence (INQUISIT 1)	CRISPRko, CRISPRi	resistor			
CREBBP*	High confidence (INQUISIT 1)	CRISPRko	resistor		Small molecule	PRI-724
IRF1*	TWAS	CRISPRi	sensitizer	Increased or decreased expression^55^		
PRMT7*	TWAS	CRISPRi	resistor	Increased expression^40^	Small molecule	SGC3027, DS-437
*CDKAL1*	High confidence (INQUISIT 1)	CRISPRko	resistor		Antibody	
*HSPA4*	High confidence (INQUISIT 1)	CRISPRi	resistor			
*NF1*	High confidence (INQUISIT 1)	CRISPRko	resistor		Antibody	
*SMG9*	High confidence (INQUISIT 1)	CRISPRi	sensitizer		Antibody	
*SOX13*	High confidence (INQUISIT 1)	CRISPRi	sensitizer			
*TCF7L2*	High confidence (INQUISIT 1)	CRISPRi	sensitizer			
*TGFBR2*	High confidence (INQUISIT 1)	CRISPRi	sensitizer		Antibody	
*AC068831.6*	Moderate confidence (INQUISIT 2)	CRISPRko	resistor			
*AZIN1*	Moderate confidence (INQUISIT 2)	CRISPRko, CRISPRi	sensitizer			
*CLK1*	Moderate confidence (INQUISIT 2)	CRISPRko	resistor		Small molecule	MU1210, SGC-CLK-1, T3-CLK
*IL6ST*	Moderate confidence (INQUISIT 2)	CRISPRi	sensitizer		Antibody	SATRALIZUMAB
*LIN54*	Moderate confidence (INQUISIT 2)	CRISPRko	resistor			
*MAP3K11*	Moderate confidence (INQUISIT 2)	CRISPRi	sensitizer		Small molecule	CEP-1347
*ORC2*	Moderate confidence (INQUISIT 2)	CRISPRko	resistor			
*RBM8A*	Moderate confidence (INQUISIT 2)	CRISPRi	resistor			
*RP11-14D22.2*	Moderate confidence (INQUISIT 2)	CRISPRko	sensitizer			
*SEC22B*	Moderate confidence (INQUISIT 2)	CRISPRi	resistor		Antibody	
*SOX4*	Moderate confidence (INQUISIT 2)	CRISPRko, CRISPRi	resistor			
*SP3*	Moderate confidence (INQUISIT 2)	CRISPRi	sensitizer			
*SRSF9*	Moderate confidence (INQUISIT 2)	CRISPRko	resistor			
*TFAP4*	Moderate confidence (INQUISIT 2)	CRISPRi	sensitizer			
*AC007283.5*	TWAS	CRISPRi	resistor			
*COX11*	TWAS	CRISPRi	resistor			
*DDA1*	TWAS	CRISPRi	resistor			
*LMO4*	TWAS	CRISPRko	resistor			
*MAEA*	TWAS	CRISPRko	resistor		Antibody	
*NPAT*	TWAS	CRISPRi	resistor			

^a^CTL screen hits, with validated genes indicated*.

^b^Direction of gene expression associated with breast cancer risk allele, from previous studies.

^c^Target tractability assessments from the Open Targets Platform^56^.

^d^Inhibitor data retrieved from the Open Targets Platform.

### Validation of candidate CTL evasion genes

We prioritized six genes for validation based on (i) high-confidence computational predictions by INQUISIT or TWAS, (ii) known regulation by breast cancer risk variants, and (iii) with known functional relevance in immune modulation pathways. To validate a subset of the 33 candidate breast cancer CTL genes identified in these screens, we performed single-gene perturbation experiments with CRISPRko and CRISPRi. The subset included genes predicted with high confidence by INQUISIT (*CFLAR, CREBBP, ATF7IP* and *CASP8*) or from TWAS (*IRF1* and *PRMT7*). In addition to these six genes, we included two positive control genes (*IFNGR2* and *JAK2*) in validation assays.

**Fig. 2:**
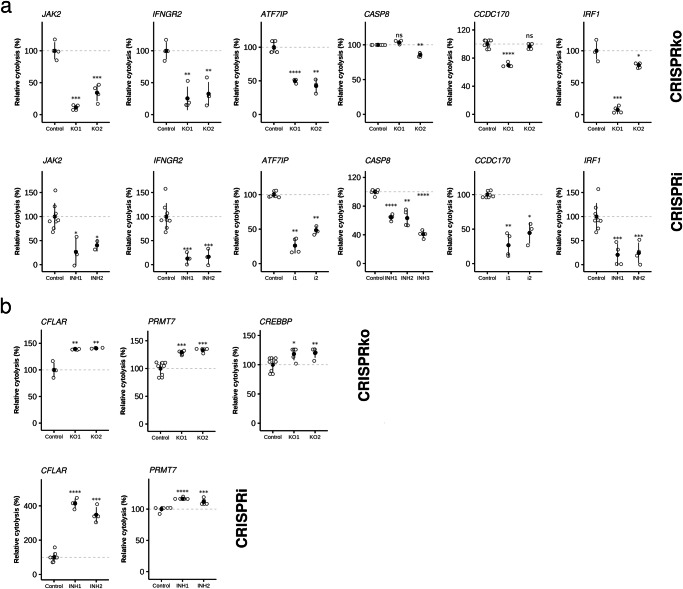
Validation of target cell cytolytic activity following inhibition or knockout of CTL evasion genes. **a** Inhibition or knockout of the positive control genes, *IFNGR2* and *JAK2*, and selected sensitizer genes rendered MCF7 cells more resistant to the HER2-restricted T cells (knockout lines shown on top row, inhibition lines along bottom row). **b** Inhibition or knockout of selected resistor genes rendered MCF7 cells more sensitive to HER2-restricted T cells. Filled circles denote sample means and whiskers represent standard deviations. Significance was computed with two-sample *t*-tests, using Benjamini–Hochberg correction for multiple testing. All experiments comprised *n* > 3. (ns = non-significant, ^*^P < 0.05, ^**^P < 0.01, ^***^P < 0.001, ^****^P < 0.0001).

To perform the single gene validation, we infected MCF7 with the top two scoring CRISPRko or CRISPRi sgRNAs from the custom libraries (except for *CASP8* which did not score in the CRISPRi screen, and so we re-designed the CRISPRi sgRNAs), followed by puromycin selection for 7 days. Gene perturbation was assessed by western blotting (Fig. S5), and/or quantitative real-time Polymerase Chain Reaction (PCR; Fig. S6). On day 7, the lines were pretreated for 24 h with 10 ng/ml IFNγ and co-cultured with T cells at an E:T ratio of 1:1 while their growth was monitored by xCELLigence. The rate of cytolysis was monitored until 70% MCF7 lysis was achieved upon exposure to HER2-restricted T cells. The relative cytolysis in the xCELLigence assay was determined relative to cells infected with a non-targeting control guide (sgRFP). As expected, inhibition or knockout of the positive control genes, *IFNGR2* and *JAK2*, rendered MCF7 cells more resistant to the HER2-restricted T cells (Fig. 2). We validated the role of *ATF7IP, CASP8* and *IRF1* as sensitizers, and of *CFLAR, CREBBP* and *PRMT7* as resistors. CRISPRi did not knock down *CREBBP* expression, but the CRISPRko results validated it as a resistor (Fig. 2).

**Fig. 3:**
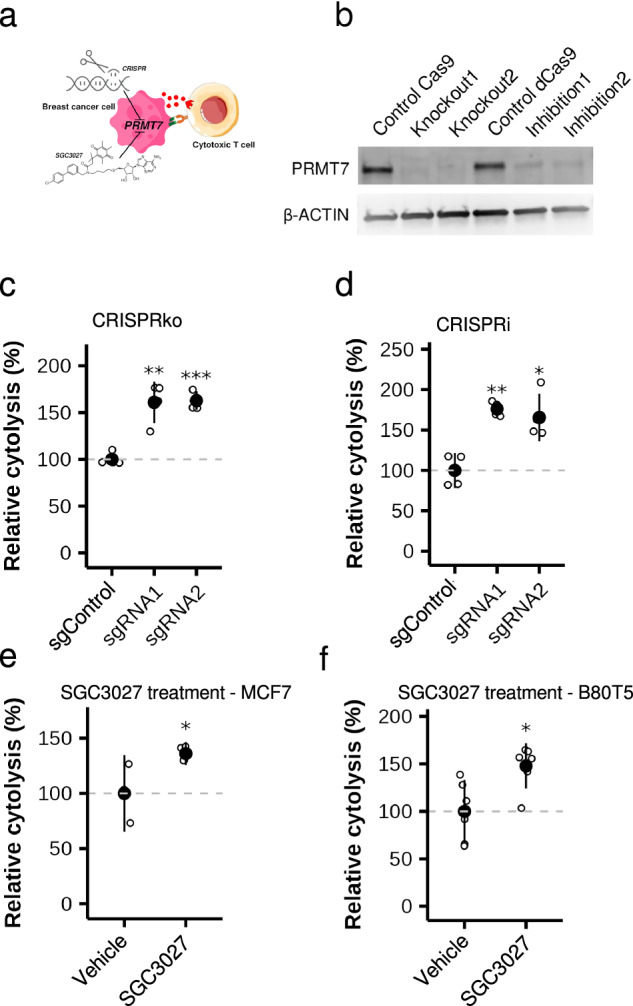
Effect of inhibition of PRMT7 activity in tumor cells on T cell killing. **a** Genetic perturbation and pharmacological inhibition of PRMT7 altered response to T cell killing**. b** Depleted PRMT7 protein levels in independent B80T5 lines upon CRISPRko and CRISPRi editing. Relative cytolysis (compared to cells with non-targeting control guide) in B80T5 following CRISPRko. The full blot is shown in Fig. S5. (**c**) or CRISPRi (**d**). PRMT7 inhibition with SGC3027 enhanced T cell-mediated killing of both MCF7 (**e**) and B80T5 (**f**). Filled circles denote sample means and whiskers represent standard deviations. Significance was computed with two-sample *t*-tests, using Benjamini–Hochberg correction for multiple testing. All experiments comprised *n* > 3. (^*^P < 0.05, ^**^P < 0.01, ^***^P < 0.001).

To further explore the role of PRMT7 in immune evasion, we tested whether its disruption also sensitizes “normal”, breast cells to T cell killing (Fig. 3a). We repeated PRMT7 CRISPR knockout and inhibition in the TERT-immortalized breast cell line B80T5, achieving >90% reduction in protein levels (Fig. 3b). Both perturbations resulted in a ~1.5-fold increase in T cell–mediated lysis (Fig. 3c, d). Although derived from normal breast tissue, we have found that B80T5 exhibits extensive copy number alterations (2605 CNVs); 88% of the total copy number changes represented amplifications (2175 gains (three to five copies) and 138 high level-gains (six to eight copies), respectively), indicating that mutations have occurred in this cell line (2copyLRR = −0.215 - data not shown). These findings indicate that PRMT7 may regulate CTL-mediated killing in cellular contexts beyond Luminal breast cancer.

### PRMT7 pharmacological inhibition in vitro

Notably, inhibition of PRMT7 with a selective small molecule inhibitor, SGC3027, can sensitize melanoma to immune checkpoint blockade^29^. We investigated the effect of PRMT7 pharmacological inhibition with SGC3027 in our co-culture assay system. Following pre-treatment of target MCF7 or B80T5 (18 h), inhibition of PRMT7 significantly increased the rate of T cell-mediated cytolysis (1.2–1.5-fold, *P* < 0.05; Fig. 3e, f). These results demonstrated that genetic and pharmacological inhibition of PRMT7 enhances the degree of immune killing in multiple breast cell contexts.

### Immune signatures in clinical samples and patient outcomes

**Fig. 4:**
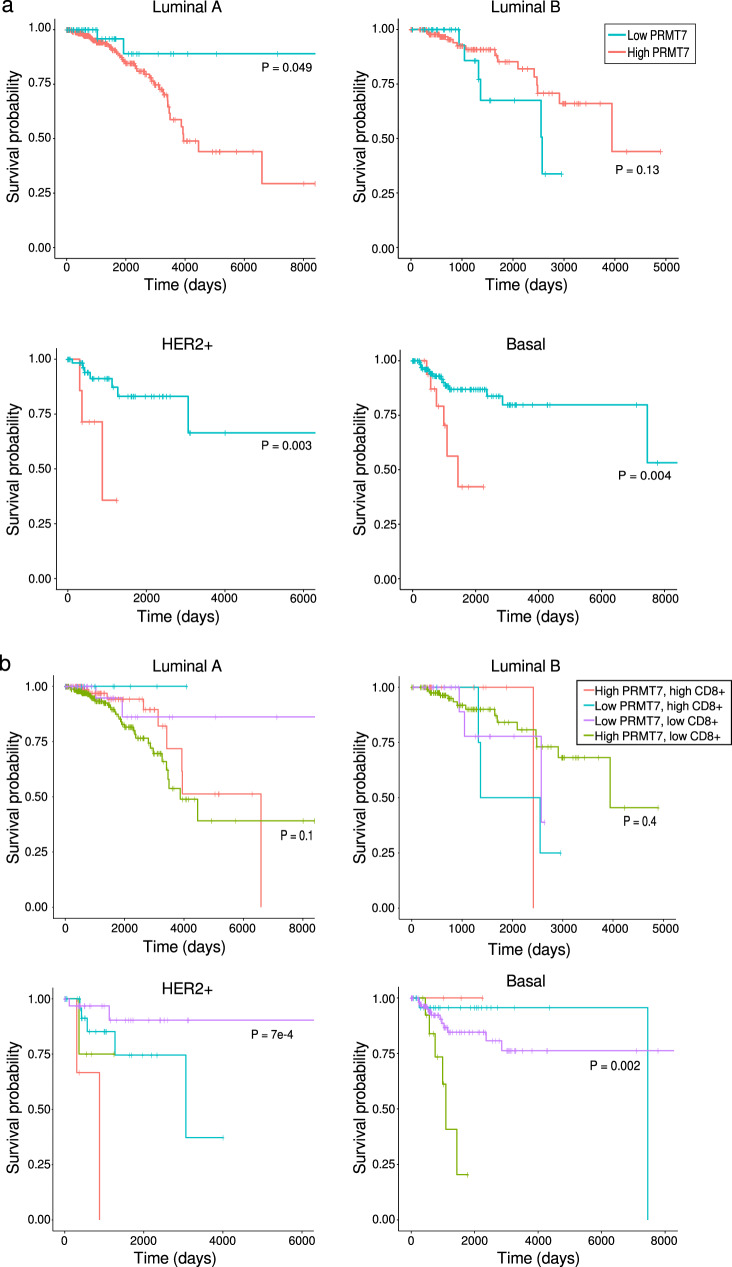
Association of *PRMT7* expression with breast cancer patient survival in the TCGA cohort. **a** High *PRMT7* expression is significantly associated with reduced survival in patients with Luminal A, HER2+ and Basal tumors. **b** *PRMT7* expression, together with the degree of CD8+ T-cell infiltration, is associated with subtype-specific survival outcomes. *P* values were calculated using log-rank tests.

In order to determine which subtypes of breast cancer might be most amenable to PRMT7 inhibition, we examined associations between gene expression and breast cancer patient outcomes. In Luminal A, HER2+, and Basal cancers (*N* = 499, 73, and 168, respectively) from The Cancer Genome Atlas (TCGA) cohort, we observed significantly reduced overall survival (OS; all nominal *P* < 0.05) in cases with elevated *PRMT7* expression (Fig. 4a). Adjusting for age and stage in a Cox Proportional Hazards model revealed a significant effect on reduced OS for patients with with Basal tumors for the highest compared to lowest tertile *PRMT7* expression (HR = 3.69, 95% CI 1.19–11.42, nominal *P* = 0.02). Consistent trends were detected for Progression-Free Interval in TCGA patients, and for OS and Relapse-Free Survival in the METABRIC (Molecular Taxonomy of Breast Cancer International Consortium) cohort for Luminal A, HER2+, and Basal cancers, although they only reached statistical significance in the Luminal A analysis (Fig. S7). Interestingly, high *PRMT7* expression was significantly associated with improved overall and relapse free survival of patients with Luminal B tumors in the METABRIC cohort (*P* < 0.025), suggesting a potential context-specific role.

We hypothesized that patient outcomes may be dependent on the interaction between *PRMT7* expression and levels of infiltrating CD8+ cells. In HER2+ tumors, despite relatively small numbers in each stratum, the combination of high *PRMT7* expression and high CD8+ infiltration was associated with the poorest survival (log-rank *P* = 7 × 10^−4^; Fig. 4c). In contrast, high PRMT7 expression together with low CD8+ infiltration in Basal tumors was associated with the worst outcomes (log-rank *P* = 0.002; Fig. 4c). A similar trend was observed in Luminal A tumors, where high PRMT7/low CD8+ was correlated with shorter survival, although this did not reach statistical significance (log rank *P* = 0.1). We next examined the relationship between expression of *PRMT7* and immune signatures in breast tumor samples using data from TCGA^30^, where *PRMT7* levels were negatively correlated with infiltrating CD8+ levels in Luminal A tumors (*ρ* = −0.25, *P* = 1.12 × 10^−8^; Fig. S7d). The associations with HER2+ or Basal tumors were not significant (*P* = 0.15 and 0.49, respectively), although only 82 and 188 samples were available for this analysis. These findings suggest that the prognostic effect of PRMT7 may depend not only on tumor subtype but also on the immune context, with distinct interactions between PRMT7 expression and cytotoxic T-cell infiltration in different molecular backgrounds.

### Prmt7 perturbation in vitro and in vivo

**Fig. 5:**
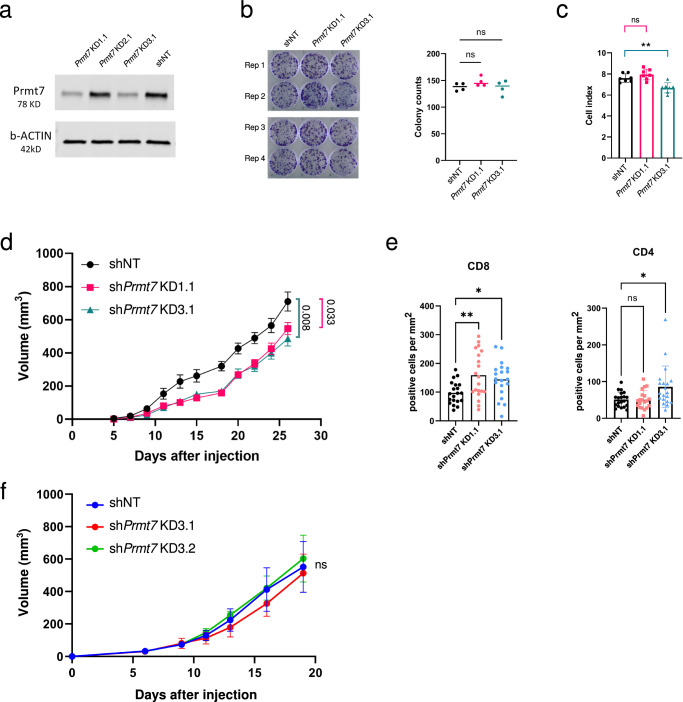
In vivo validation of *Prmt7* depletion in 4T1 orthotopic models. **a** Western blot of *Prmt7* knockdown lines and *Prmt7* wild-type control line. **b** In vitro proliferation was assessed using colony formation. Replicate colony counts per well were analyzed using One-way ANOVA. **c** Real time cell growth was measured using the xCELLigence system and the significance of cell index at experimental endpoint was quantified using ANOVA. **d** In vivo tumor volume over time for transplanted 4T1 cells transduced with sh*Prmt7* KD1.1, sh*Prmt7* KD1.3, and non-targeting control shRNA (shNT) implanted into BALB/c mice (*n* = 8). Data were presented as mean ± s.e.m and analyzed by unpaired, two-sided Student’s *t*-tests. **e** Immunohistochemistry analysis to evaluate levels of CD8+ and CD4+ T cell infiltration. Cell counts per area (mm^2^) were compared using one-way ANOVA with Dunnett’s multiple correction test. **f** Tumor growth of *Prmt7*^null^ clones in immunocompromised mice (*n* = 6–7). ^*^P < 0.05, ^**^P < 0.01).

To examine the tumor-intrinsic role of Prmt7 in vitro and in vivo, we generated *Prmt7*-deficient 4T1 murine triple negative mammary carcinoma cells. We used short hairpin RNA (shRNA)-mediated knockdown (KD) to avoid Cas9-dependent tumor rejection in immunocompetent hosts^31^. Western blot showed successful Prmt7 protein KD with shPrmt7 KD1.1 and KD3.1 (Fig. 5a). No growth effect was observed using colony formation assays (Fig. 5b), while a significant effect on in vitro 2D cell growth was observed for only the shPrmt7-KD3.1 clone (*P* = 0.003) using the xCELLigence Real Time analyzer (Figs. 5c and S8). We implanted control and *Prmt7*-deficient 4T1 cells (clones shPrmt7 KD1.1 and shPrmt7 KD3.1) into mammary fat pads of syngeneic BALB/c mice and monitored tumor growth. Relative to control tumors, both KD clones exhibited significantly reduced volume (*P* = 0.033 and 0.008, Figs. 5d and S9). These data suggest that *Prmt7* depletion reduces tumor growth in the 4T1 breast cancer model. We examined tumors recovered from immunocompetent hosts using immunohistochemistry to evaluate the levels of T cell infiltration. We observed significantly increased levels of CD8+ cells infiltration for both clones (*P* < 0.01, Analysis of Variance (ANOVA)), and increased levels of CD4+ cells in shPrmt7 KD3.1 tumors (Figs. 5e and S10), indicating changes to the tumor microenvironment after disrupting Prmt7 function in vivo. To further test whether the diminished growth of *Prmt7*^null^ tumors was dependent on T cell activity, we injected two *Prmt7*-deficient 4T1 cell lines into immunocompromised BALB/c nude mice. No difference in tumor size was observed at endpoint (Figs. 5f and S11), suggesting that an intact immune system is required for tumor control.

## Discussion

GWAS have been successful at identifying loci associated with breast cancer risk, with over 220 identified so far^1,2^. Most of the candidate causal variants identified through fine-mapping lie in non-coding regions, and most likely act by regulating the expression of one or more nearby genes^3^. One of the major goals of GWAS is to find target genes and proteins that might lead to new strategies for prevention or treatment. This is particularly important since several studies have shown that the likelihood of a drug getting to market is approximately doubled if it has genetic support^32,33^. However, identifying the target gene(s) at a locus, which has been achieved for fewer than 20 loci (including specific analysis showing *ATF7IP* and *CASP8* are regulated by breast cancer risk variants)^4,34^, is time consuming and does not address the issue of molecular pleiotropy: it is important to know not only which target genes are genetically regulated, but also that the impact is on a phenotype relevant to the disease or trait of interest. To date, most functional studies have focused on tumor-intrinsic processes such as proliferation and DNA repair^4^. Yet breast cancer progression is also strongly influenced by the immune microenvironment, raising the possibility that risk alleles may contribute to disease through modulation of tumor–immune interactions. The T cell-mediated tumor cell evasion screens presented here extend screening approaches to systematically identify candidate risk genes that affect not only tumor-intrinsic pathways but also immune surveillance.

Of the 33 candidate breast cancer genes we identified in our CTL screens, 18 have not been previously reported in published tumor immunity-associated functional screens^35^. Consistent with our findings, *CASP8* has been shown to act as a sensitizer, and *CFLAR* and *CREBBP* as resistors^26^. In contrast, *ATF7IP* has been previously shown to be a resistor in a murine melanoma model^36^, and *IRF1* and *PRMT7* have been variably found to be resistors or sensitizers^26,29^. Lawson et al.^26^ carried out genome-wide CTL co-culture screens using two murine triple-negative mammary carcinoma cell lines and identified *IRF1, CFLAR, CREBBP, ATF7IP, CASP8* and *PRMT7*, although not always in the same direction as we found in our screens in the Luminal A cell line, MCF7. These findings suggest that cell type-specific and histological subtype differences influence tumor-immune phenotypes.

*CASP8* and *CFLAR*, co-located at the chromosome 2q33.1 breast cancer risk locus, were prioritised as high-confidence GWAS target genes^3^. CASP8 promotes apoptosis but also shapes adaptive immune responses through effects on lymphocyte activation and macrophage polarisation^37^. In contrast, CFLAR resists T cell cytotoxicity by blocking death receptor signaling through FAS and TRAIL^38^, mechanisms commonly used by cytotoxic T cells and Natural Killer cells. High CFLAR expression may therefore provide tumors with a selective advantage under immune pressure, and indeed, CFLAR overexpression enables tumor growth in immunocompetent mice by suppressing Fas-mediated apoptosis^39^. Consistent with these opposing functions, CASP8 and CFLAR were detected as a sensitizer and a resistor, respectively, in our screens. An open question is whether GWAS signals at the 2q33.1 locus map to elements that differentially regulate *CASP8* and *CFLAR* expression, thereby shifting the balance between immune sensitivity and resistance.

*PRMT7* emerged from a transcriptome-wide association study of breast cancer susceptibility, where risk alleles were associated with increased predicted expression^40^. Consistent with this oncogenic effect, high *PRMT7* expression was linked to reduced survival in Luminal A, HER2+, and Basal subtypes in clinical cohorts. While our in vitro co-culture system relied on response to the experimental HER2 antigen, PRMT7’s role in immune evasion may extend beyond this context. In support of this, we validated functional effects in multiple systems. Genetic deletion and pharmacological inhibition enhanced T cell-mediated cytolysis of B80-T5 and MCF7 cells in vitro, while evasion effects were consistent within in vivo settings using the murine triple-negative mammary carcinoma 4T1 model. In these tumors, Prmt7 loss was associated with increased CD8+ T cell accumulation, despite no clear evidence that PRMT7 directly regulates immune cell trafficking. This effect was mirrored in clinical samples, in which high *PRMT7* expression and low CD8+ tumor infiltration were found to be associated with reduced survival in triple-negative Basal tumors. We note that the association between high expression and high CD8+ infiltration in HER2+ tumors may reflect divergent regulatory roles for PRMT7, potentially through induction of immune suppression.

As a histone methyltransferase, PRMT7 modulates chromatin function and gene expression, potentially influencing different downstream pathways across subtypes. Therefore, its downstream effects may primarily be shaped by the microenvironment or dominant signaling context of each subtype. For example, in Luminal A tumors (estrogen receptor-positive), PRMT7 might repress interferon response genes or antigen-processing components, contributing to immune exclusion. In contrast, in triple-negative Basal tumors, characterized by intrinsic immune activity, PRMT7 may primarily enhance tumor cell-intrinsic resistance to cytotoxicity (e.g., via modulation of apoptosis regulators or stress response genes), rather than directly altering lymphocyte infiltration. We speculate that PRMT7 promotes immune evasion through epigenetic repression of antigen-processing and presentation pathways, thereby reducing tumor immunogenicity. Future flow cytometry, multiplex immunohistochemistry, and single-cell transcriptomics studies will test whether PRMT7 inhibition alters T cell effector function (e.g., IFNγ, granzyme B) in the tumor microenvironment. Collectively, these findings identify PRMT7 as a novel immuno-oncogenic driver in breast cancer, and suggest that targeted inhibition, alone or in combination with immune checkpoint blockade, may enhance antitumor immunity and overcome therapeutic resistance.

To interpret these findings in the context of breast cancer immunogenetics, it is also important to consider technical aspects of the HER2 antigen-specific T cell assays we employed. The HER2 T Cell Receptor (TCR) used in this study was derived from a well-characterized allo-restricted HER2₃₆₉–₃₇₇ epitope^41^, whose specificity has been established previously and confirmed here by the preferential killing of HER2-TCR CTLs over mock-transduced T cells. Although MCF7 cells are not HER2-amplified, our cytolysis assays showed they are markedly more sensitive to killing by HER2-restricted CTLs than HER2^high^ SKBR3 cells. This finding suggests that antigen availability from HER2 expression alone is not the primary dominant determinant of CTL susceptibility. Instead, factors such as HLA-A2 expression, peptide processing efficiency, and immune checkpoint ligand levels may be more critical in shaping CTL recognition. Importantly, validation of the T cell evasion phenotype following *PRMT7* knockout in additional HER2^moderate^ cell line models (e.g., B80-T5) supports the idea that PRMT7 may be a general regulator of antigen presentation. An ER+ mammary carcinoma cell line, B6BC, was reported recently^42^, which may enable further subtype-specific in vivo validation of the hits from our screens and pre-clinical studies for targeted therapy development. Although we cannot completely rule out off-target effects, the consistent enhancement of CTL cytolysis upon PRMT7 inhibition or knockout across multiple cell lines strongly supports a role for PRMT7 in antigen-specific immune evasion.

We observed a modest correlation (*ρ* = 0.25) between gene-level effects in CRISPRi and CRISPRko screens. This likely reflects differences in gene dosage sensitivity, chromatin context, and protein stability. CRISPRi typically results in partial knockdown, while CRISPRko can yield complete loss of function. Some genes may exhibit threshold-dependent effects, where partial versus complete loss produces distinct phenotypes. Furthermore, CRISPRi may only affect a subset of isoforms or result in off-target effects in the case of bi-directional promoters. These factors highlight the need for complementary approaches in future studies.

Finally, we did not directly examine whether breast cancer risk-associated variants regulate *PRMT7* expression. The gene was selected based on predictions from a transcriptome-wide association study in normal breast tissue, which aggregates the effects of multiple Single Nucleotide Polymorphisms (SNPs). As such, identifying specific causal variants for experimental follow-up remains challenging, and the most appropriate cellular models for such analyses are not clear. Future genetic studies with larger sample sizes may identify candidate causal variants with subtype-specific associations and therefore enable direct functional evaluation of gene regulatory mechanisms.

In summary, our findings highlight the value of pooled functional screens in identifying genes associated with cancer hallmarks. These findings extend the relevance of GWAS loci beyond proliferative or DNA repair phenotypes and underscore the importance of immuno-genetic mechanisms in breast cancer risk. Such insights could ultimately guide prevention strategies or patient stratification for immunomodulatory therapies, aligning with the broader goal of genetically informed intervention.

## Methods

### Identification of appropriate breast cell lines for the CTL killing assay

To establish the CTL killing assay with breast cell lines, we first determined their classical HLA types. Breast cell lines were genotyped at the Genomic Core Facility of Erasmus University Rotterdam (Netherlands) using the Illumina Global Screening SNP Array. We used the SNP2HLA method^43^ to impute classical HLA types based on the observed genotypes. We confirmed HER2 surface protein expression levels in HLA-A2-positive breast carcinoma cell lines, MCF7 (ER+), SKBR3 (HER2 amplified), and the immortalised normal breast cell line B80T5 (ER-)^4,44^ by flow cytometry. Briefly, cells were seeded in 6-well plates at a density of 3 × 10^5^ cells per well and cultured overnight. Cells were stimulated with human IFNγ (10 ng/ml, Sigma-Aldrich, Cat# SRP3058) for 24 h. Cells were washed with PBS and dissociated from plates using TrypL^TM^ Select Enzyme (Gibco, Cat# 12563011). To determine cell surface expression levels of HER2 and HLA, cells were stained with anti-HER2 (clone 24D2, BioLegend, PE anti-human CD340 (erbB2/HER-2) Antibody, Cat# 324406) and anti-HLA (clone W6/32, PE anti-human HLA-A,B,C Antibody, BioLegend, Cat# 311405) antibodies, respectively. Live and dead cells were distinguished using a Zombie Aqua^TM^ Fixable Viability Kit (BioLegend, Cat# 423102). Cells were fixed with Cytofix^TM^ Fixation Buffer (BD, Cat# 554655) and analyzed on BD LSRFortessa^TM^ Cell Analyzer.

B80T5 and MCF7 cells were cultured in RPMI-1640 (Gibco) supplemented with 10% fetal bovine serum (FBS, Gibco). SKBR3 and MDA-MB-231 cells were cultured in Dulbecco's Modified Eagle Medium (DMEM; Gibco) supplemented with 10% FBS, 20 mM HEPES (Gibco) and 1 mM sodium pyruvate (Sigma-Aldrich). All cell lines were maintained in a humidified incubator at 37 °C with 5% CO_2_ and tested regularly for mycoplasma infection.

### Isolation of peripheral blood mononuclear cells

In order to identify an HLA-matching donor as a source of T cells, blood samples from healthy donors were collected with donors’ informed consent according to the requirements of the Human Research Ethics Committee of QIMR Berghofer (P3416), and research involving human research participants was performed in accordance with the Declaration of Helsinki. The donors were HLA-typed (Victorian Transplantation & Immunogenetics Services, Australian Red Cross Lifeblood, Melbourne, Australia) to identify donors, and peripheral blood mononuclear cells (PBMCs) from a healthy female HLA-A2 donor were isolated using Ficoll separation. Whole blood from EDTA tubes (BD, Cat# 366643) was centrifuged at 500 × *g* for 10 min at room temperature with no brake to remove plasma, the remaining blood was mixed at a 1:1 ratio with room temperature RPMI-1640 medium, layered over Ficoll-Paque PLUS (GE, Cat# 17144002) or Lymphoprep™ (Stemcell Technologies, Cat# 07851) in SepMate tube (Stemcell Technologies, Cat# 85450), and spun at 1200 × *g* for 10 min at room temperature. The PBMC layer was collected and washed with RPMI-1640. Cells were counted, centrifuged again and resuspended in freezing medium (90% FBS + 10% DMSO) and stored in cryogenic vials in liquid nitrogen.

### Generation of HER2-T cell receptor expressing T cells

To establish an antigen-specific CTL killing assay of breast cell lines, we generated HER2-restricted TCR-expressing T cells. We used a lentivirus-based allo-restricted TCR-expressing construct for the HER2-derived peptide 369 (HER2_369_)^41^. The TCR was modified within the constant regions to enhance stability and expression levels by replacing selected amino acids with the murine counterparts^45^. The TCR α- and β-chain sequences were then synthetically introduced into a lentivirus plasmid as one transcript using a cleavage protein to produce the α- and β-chains in equal ratios driven by the hEF-1α promoter (Biosettia Inc., San Diego). This construct (pLV-EF1a-HER2-1-TCR1), along with plasmids pMDL, pVSV-G and pREV, was transfected into HEK293T packaging cells. Lentiviral supernatant was harvested after 24-, 48- and 72-h incubation in DMEM containing 10% FBS and passed through a 0.45-μm Milli-hex filter before ultra-centrifugation (10,500 rpm, >6 h at 4 °C).

To activate the T cells in PBMCs, 24-well plates (Falcon, Franklin Lakes, NJ) were coated with a mixture of anti-Human-CD3 (clone OKT3, eBioscience, Cat# 05121-25-500) and CD28 (clone CD28.2, eBioscience, Cat# 10311-25-500) mAbs at 0.5 µg/ml. PBMCs were plated at 2 × 10^6^ per well to the CD3/CD8 coated plate in AIM V™ complete media (AIM V™ SFM, Gibco Ca#: 0870112DK + 5% CTS™ Immune Cell SR, Gibco Cat# A2596101) supplemented with 300 IU/ml rIL-2. Eighteen hours after activation, PBMCs were transduced with the HER2-TCR expressing lentivirus using the spin infection method on CD3/CD28 and Retronectin (16 μg/ml, Takara, Cat# T100B) coated plates with the addition of 4 μM TBK1/IKKɛ complex inhibitor BX-795 (Selleck Chemicals, Cat# S1274) to enhance lentiviral delivery^46^. Six hours after infection, the media was adjusted to 1.3 μM BX-795. After a further 60 h, the media was changed to RPMI-1640 with 10% heat-inactivated FBS, and the cells were plated into G-Rex^®^ 6 Well plates (Wilson Wolf, Cat# 80240 M) as per manufacturer’s instructions for large-scale expansion before harvesting on day 13. A single batch of HER2-TCR T cells (henceforth called HER2-restricted T cells) was generated, expanded and cryo-preserved for all screens and subsequent validations. The HER2-TCR infection rate was determined by flow cytometry in CD3+, CD4+ and CD8+ cells with a TCR Vβ8 antibody, in comparison with mock-transduced PBMCs. The panel included antibodies for TCR Vβ8 FITC (clone 56C5.2, Beckman Cat# IM1233), CD3 BV711 (clone SK7, BD Pharmingen™ Cat# 740832), CD4 (clone SK3, BD Pharmingen™ Cat# 612748), CD8 (clone SK1, BD Pharmingen™ Cat# 565310) and viability dye Sytox Blue (Invitrogen™, Cat# S34857) and was analyzed on BD LSRFortessa™ Cell Analyzer.

### T cell cytotoxicity assay

In order to perform the pooled CRISPR screens in breast cells, we adopted the two-cell type assay previously described to identify essential genes for immunotherapy^23,26,47^. MCF7, SKBR3, MDA-MB-231, and B80T5 were cultured in 96-well E-Plates (Agilent) for 24 h followed by co-culturing with HLA-A2-HER2-restricted T cells on the xCELLigence RTCA instrument (Agilent) until the rate of cytolysis plateaued^48,49^. We added 100 μL of RPMI-1640 containing 10% FBS to each well, and measured the background impedance, displayed as the Cell Index. Dissociated breast cells were then seeded at a density of 2 × 10^4^ (MCF7, SKBR3 and MDA-MD-231) or 10^4^ (B80T5) cells/well of the E-Plate in a volume of 100 μL RPMI-1640 with 10% FBS media and allowed to passively adhere on the electrode surface for 30 min inside the cell culture incubator hosting the RTCA instrument. Impedance data, Cell Index, Normalized Cell Index, and Percentage Cytolysis were recorded at 15 min intervals for the duration of the experiment. The initial cell index for each well before co-culture was used to assess breast cell health status, viability and growth. When the co-culture started, HER2-restricted T (effector) cells were added to the breast (target) cells at the indicated (E:T) ratio. Percent cytolysis was determined at each time point using the xIMT software (Agilent). The rate of cytolysis was calculated for each well at every time point, using the Normalized Sample Cell Index and the Normalized Average Target Alone Control according to Eq. (1).

1 $$\% {Cytolysis}=\left(1-\frac{C{I}_{{ti}}/C{I}_{{nml}\_{time}}}{C{I}_{{TargetAlone}.{ti}}/C{I}_{{TargetAlone}.{nml}\_{time}}}\right)\times 100$$

Effector cell controls, full lysis controls and target plus mock-transduced PBMCs (T blast) controls were used for optimization of the assay. Effector cell only controls were comparable to baseline level of impedance while full lysis controls show 100% cytolysis shortly after being applied to the adherent breast cells.

To find the optimal low dose for enhancing MHC-I expression in MCF7 cells within 24 h, we titrated the concentration of IFNγ ranging from 0.1 to 100 ng/ml. The HLA-ABC level of MCF7 cells was assessed by flow cytometry (BD Pharmingen™ PE Mouse Anti-Human HLA-ABC, Cat# 565291) following 24 h treatment with different concentrations of IFNγ.

### Generation of lentiviruses

Lentiviruses were produced as previously described^4^. Briefly, sgRNAs or expression vectors were transfected along with pMD2.G (Addgene #12259) and psPAX2 (Addgene #12260) lentiviral vectors which were transfected into HEK293T packaging cells. Lentiviral supernatant was harvested after 48 h incubation in DMEM containing 30% FBS and passed through a 0.45 μm Milli-hex filter.

### Pooled sgRNA libraries

The custom CRISPRko (knockout) and CRISPRi (inhibition) screen libraries targeting candidate breast cancer risk genes were generated as described^4^. Pooled oligonucleotides were cloned into the BsmBI sites of pLeniGuide-Puro (Addgene #52963). We included sgRNAs targeting core cell essential genes to monitor screen performance, as well as genes known to confer resistance to T cell-mediated killing as phenotypic positive controls, *APLNR*, *ARID2*, *B2M*, *BBS1, CD58, CTLA4, ERAP1, ERAP2, HLA-A, IFNGR1, IFNGR2, IRF2, IRF8, JAK1, JAK2, NLRC5, PBRM1, PDCD1, CD274, STAT1, TAP1, TAP2* and *TPP2*^21,23,26,50^. One thousand negative control sgRNAs targeting the *AAVS1* locus were included. For each gene, we chose top scoring five sgRNAs designed using the CRISPick algorithm. sgRNA sequences are available in Table S1. Libraries were electroporated into NEB5α electrocompetent cells (NEB), grown overnight and plasmid DNA extracted using Qiagen Maxi Prep Kit (Qiagen, Cat# 12162). For each library preparation, at least 1000-fold guide representation was ensured by counting surviving colonies from the serially diluted samples.

### Pooled CRISPR screens for resistance to T cell cytotoxicity

For CRISPR screens and validations, breast cell lines were transduced with Lenti-Cas9-2A-Blast (Addgene, Cat# 73310) or Lenti-dCas9-KRAB-Blast (Addgene, Cat# 89567). MCF7 cells stably expressing Cas9 or KRAB-dCas9 were established by transduction of the virus at MOI < 0.7^51^, selected in 10 μg/ml blasticidin (Gibco, Cat# A1113903) and maintained in 5 μg/ml blasticidin. MCF7 cells were transduced with CRISPRko or CRISPRi library virus at a MOI of 0.3 to ensure one guide per cell and obtain 1000 cells per sgRNA. Library infection was carried out in triplicate. Twenty-four hours after infection, cells were selected using puromycin (1 μg/ml, Gibco, Cat# A1113803) for 7 days. In order to assay for resistance to HER2-T cell cytotoxicity, library-infected MCF7 cells were cultured for 24 h with interferon-gamma (IFNγ) at 10 ng/ml followed by co-culturing with HER2-restricted T cells at an Effector (E; HER2-T cells): Target (T; MCF7) ratio of 1:1. The control group was cultured for a recovery period equal to the T cell co-culture period. A parallel xCELLigence assay was run for each screen for real-time monitoring of cytolysis. When the cytolysis rate reached 70% in the co-culture flask (MCF7+ T cells), the media was removed from both the co-culture and the unexposed controls. After three washes with PBS, the cells were maintained with blasticidin (10 μg/ml) and puromycin (1 μg/ml) for another 48 h. Cells were harvested and genomic DNA extracted using NucleoSpin Blood XL kit (Clontech). Libraries were prepared and sequenced as previously described^4^.

### Pooled screen analysis

Raw sequencing reads were processed by trimming adaptors and vector sequences using *cutadapt* v1.13 with parameter ‘*-g TTGTGGAAAGGACGAAACACCG*’. sgRNA barcodes were counted using *MAGeCK* (v0.5.9.4) and the significance of treatment-dependent abundances calculated using maximum-likelihood estimation implemented in the MLE module, with 10 rounds of permutation (each round corresponding to 2 × gene number), correcting for MCF7 gene copy number using CNV array data from the Broad Institute Cancer Cell Line Encyclopedia and normalizing by counts of negative control sgRNAs. Experimental treatment conditions for MLE modeling were supplied as binary design matrices including variables for baseline, proliferation (to capture general fitness effects in culture), IFNγ response, and T cell exposure.

### Generation of stable cell lines for single gene validation

For single gene validation, two sgRNAs with the strongest effect in respective screens were cloned into BsmBI-digested lentiGuide-Puro vector (Addgene, Cat# 52963) (Supplementary Table 1). MCF7 or B80T5 cells with single gene perturbation were generated as previously described^4^. Gene perturbation was confirmed by Western blotting or qPCR. Total protein or RNA was isolated from these cells at the comparable passage number with which the cytolysis assay was performed. Total RNA isolation was performed using the RNeasy Mini Kit (Qiagen, Cat# 74104). cDNA was synthesized using the Maxima H Minus First Strand cDNA Synthesis Kit (Thermo Scientific, Cat# K1682) and amplified using PowerUp™ SYBR™ Green Master Mix (Thermo Scientific, Cat# A25742). The mRNA levels for each sample were measured in technical triplicates for each primer set, along with three housekeeping genes (*ACTIN*, *GUSB*, and *PUM1*; Supplementary Table 2). Experiments were performed using an ABI ViiA(TM) 7 or ABI Q5 QuantStudio System (Applied Biosystems), and data processed using ABI QuantStudio™ Software (Applied Biosystems). The average Cт of target genes was compared with the geometric mean of three housekeeping genes to calculate ΔCт. Relative quantitation of each target gene was normalized to the corresponding sgRFP control line using the comparative Cт method (ΔΔCт).

Whole cell lysates were prepared with RIPA buffer containing protease inhibitors (Invitrogen) as per manufacturer’s instructions before quantification with Bicinchoninic Acid assay (Thermo Scientific). Samples were reduced and boiled prior to loading 20 μg per lane onto precast 4–15% Mini-Protean TGX polyacrylamide gels (BioRad Laboratories Inc, Cat# 4561086), followed by transfer onto low-fluorescence PVDF membranes with a Trans-blot Turbo Transfer System (BioRad Laboratories Inc). After blocking for 1 h in Intercept (TBS) Blocking Buffer (LI-COR Biosciences), membranes were probed overnight at 4 °C with primary antibodies against the respective target: ATF7IP (Sigma-Aldrich, Cat# HPA023505), CFLAR (Cell Signaling Technology, Cat# 56343), CREBBP CBP (D6C5) (Cell Signaling Technology, Cat# 7389), IFNGR2 (OTI1C2) (Origene Technologies Inc, Cat# TA506734S), IRF1 (BioRad Laboratories Inc, Cat# VPA00801) and PRMT7 (D1K6R) (Cell Signaling Technology, Cat# 14762). Fluorescent detection and imaging on the LI-COR Odyssey CLx instrument (LI-COR Biosciences) was carried out the next day. All targets were normalized to a housekeeping gene, either beta-Actin (AC-74) (Sigma-Aldrich, Cat# A2228) or Cytochrome C oxidase (COXIV; LI-COR Biosciences, Cat# 926-42214), using the ImageStudio software package.

### In vitro PRMT7 inhibitor studies

Background impedance was measured by adding 100 µL of RPMI-1640 with 10% FBS media to each well of the xCELLigence E-96 plate. MCF7 or B80T5 were seeded at a density of 2 × 10^4^ or 10^4^ cells/well in a volume of 100 µL RPMI-1640 with 10% FBS media and allowed to passively adhere on the electrode surface. IFNγ was added to the media to achieve the final concentration of 10 ng/mL. After 6 h, 10 µM SGC3027 (Sigma-Aldrich, Cat# SML2343) was added, or 0.1% DMSO as a negative control. Eighteen hours after adding SGC3027, the culture medium was refreshed (removing IFNγ, SGC3027 and DMSO) and T cells were added at an E:T ratio of 1:1. Cytolytic rates for samples exposed to T cells were normalized using the cell index values from unexposed controls.

### Survival analysis in clinical cohorts

Bulk RNA-seq, molecular subtype and survival data were obtained from METABRIC^52^ and TCGA^30^. Patients with multiple tumor samples were excluded. The prognostic association of PRMT7 gene expression was assessed using Kaplan–Meier survival analysis. To evaluate the joint effect of *PRMT7* expression and CD8+ T-cell infiltration, immune cell fractions in TCGA tumors were quantified as previously described^30,53^. Patients were stratified into four subgroups based on high or low *PRMT7* expression and high or low CD8+ T cell infiltration, and the differences in survival tested using the log-rank test. All survival analyzes, including determining optimal cut-off points, were performed using the R packages “*survival*” and “*survminer*”. Immune cell infiltration data for TCGA tumors were obtained through the TIMER2 portal^54^.

### Generation of Prmt7^null^ 4T1 cell lines

Lentiviral SMARTvector shRNAs (mCMV promoter) targeting murine *Prmt7* (sh*Prmt7*#1: 5′-ATGCAGTGTGTGTACTTCC-3′, sh*Prmt7*#2: 5′-GGATGGAGTATCAGCTCAC-3′ and sh*Prmt7*#3: 5′-ACAACCTGTACTTCTGGTA-3′) as well as a non-targeting control (shNTC: 5′-CACACAACATGTAAACCAGGGA-3′) were amplified from bacterial glycerol stocks (SMARTvector Lentiviral shRNA Set of three Glycerol stocks, Dharmacon). Lentiviral particles were packaged in HEK293T cells by co-transfection of shRNA constructs with psPAX2 (Addgene, Cat# 12260) and pMD2.G (Addgene, Cat# 12259). After 48 h of incubation in DMEM supplemented with 30% FBS, the lentivirus was collected by filtration through a 0.45 μm filter, aliquoted and stored at −80 °C. 4T1 cells were plated at 2.5 × 10^5^ per well of a 6-well plate in growth media (RPMI-1640 with 10% FBS) plus 8 µg/ml polybrene and infected with each shRNA lentivirus. Twenty-four hours post-transduction, 4T1 cells were cultured in growth media with 10 µg/ml puromycin for 3–5 days to select for stable knockdown lines. Prmt7 knockdown was assessed via western blot, probing with PRMT7 (D1K6R) antibody (New England Biolabs, Cat# 14762) and lines with the best knockdown were used for in vivo studies.

### Mouse studies

Seven- to nine-week-old female BALB/c mice were kept with access to food and water in a pathogen-free environment. For tumor injection, mice were anaesthetised with isoflurane, induced at a vaporizer concentration of 2.0–3.5% in oxygen with a flow rate of 0.5–1 L/min. 5 × 10^4^ non-targeting shRNA (shNT) or sh*Prmt7* (sh*Prmt7* KD1.1 and sh*Prmt7* KD3.1) 4T1 cells, prepared in 30 μL FBS-free RPMI-1640, were injected into the right fourth mammary fat pads (MFP) of female BALB/c mice to examine whether *Prmt7* deficiency affects tumor growth in vivo. Each tumor was measured manually every 2–3 days beginning on day 5 after injection. Tumor volume was calculated with the formula (length × width^2^)/2. The percentage change in mouse body weight was compared to the day when the challenge started. Mice were euthanised by CO₂ inhalation or cervical dislocation, in accordance with institutional animal ethics guidelines. All animal work was conducted in accordance with the National Health and Medical Research Council guidelines under the approval of the QIMR Berghofer Animal Ethics Committee (A2308-614), where tumor sizes did not exceed the maximum ethically permitted volume (1000 mm^3^). Statistical tests were performed using GraphPad Prism.

### Immunohistochemical staining

Infiltration levels of CD8+ and CD4+ T cells were detected by immunohistochemistry. Tissue slides were incubated with primary anti-mouse CD8a antibody (1:150, eBioscience, Cat# 14-0808) and anti-mouse CD4 antibody (1:200, eBioscience, Cat# 14-9766), followed by Rat probe and Polymer (Biocare Medical) as secondary reagent, and DAB (Biocare Medical) for staining. Five areas were selected randomly from each slide, and the CD8+ and CD4+ T cells numbers per area (mm^2^) were quantified using QuPath (version 0.5.1). Counts were compared using one-way ANOVA with Dunnett’s multiple correction.

## Supplementary information

Supplementary_Material

Supplementary_Data_1

Supplementary_Data_2

Supplementary_Data_3

## Data Availability

The datasets generated and analyzed during the current study are available in the NCBI GEO repository, GSE316985. DepMap and CCLE data were downloaded from depmap.org/portal. Gene expression data from the METABRIC study was accessed through approved access to European Genome-phenome Archive (EGA) project number EGAD00010000210.
