# Supplementary material for: CRISPR screens identify PRMT7 as a therapeutic target to enhance T cell-mediated killing in breast cancer
Source: NPJ Breast Cancer. 2026 Jan 21;12:24. doi: 10.1038/s41523-025-00888-8 (PMC12891518; doi:10.1038/s41523-025-00888-8)
Supplement: Supplementary file 1 — Supplementary_Material [file 41523_2025_888_MOESM1_ESM.pdf]

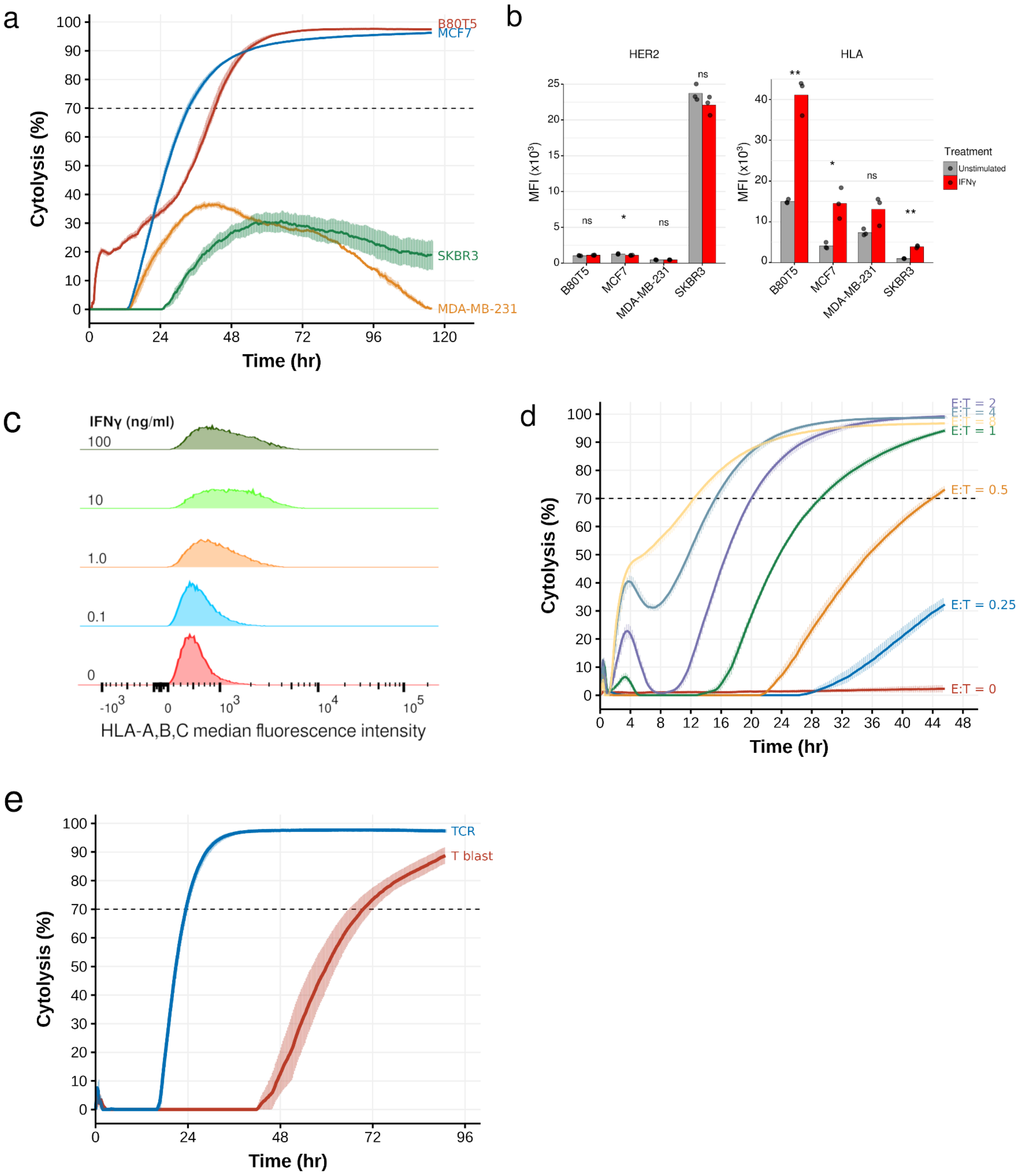

Figure S1. Optimisation of co-culture assay conditions. a) T cell-mediated cytolytic efficiency of four breast cell lines co-cultured with HER2-restricted T cells at an effector:target (E:T) ratio of 1:1. **b**) Cell surface HER2 and HLA Mean Fluorescence Intensity (MFI) in control and IFNγ-treated cells, measured by FACS. **c**) IFNγ induction of HLA-ABC in MCF7 cells. **d**) MCF7 cytolysis at increasing E:T ratios. **e**) MCF7 cytolysis is dependent on HER2 recognition by HER2-restricted T cells at E:T ratio of 1:1.

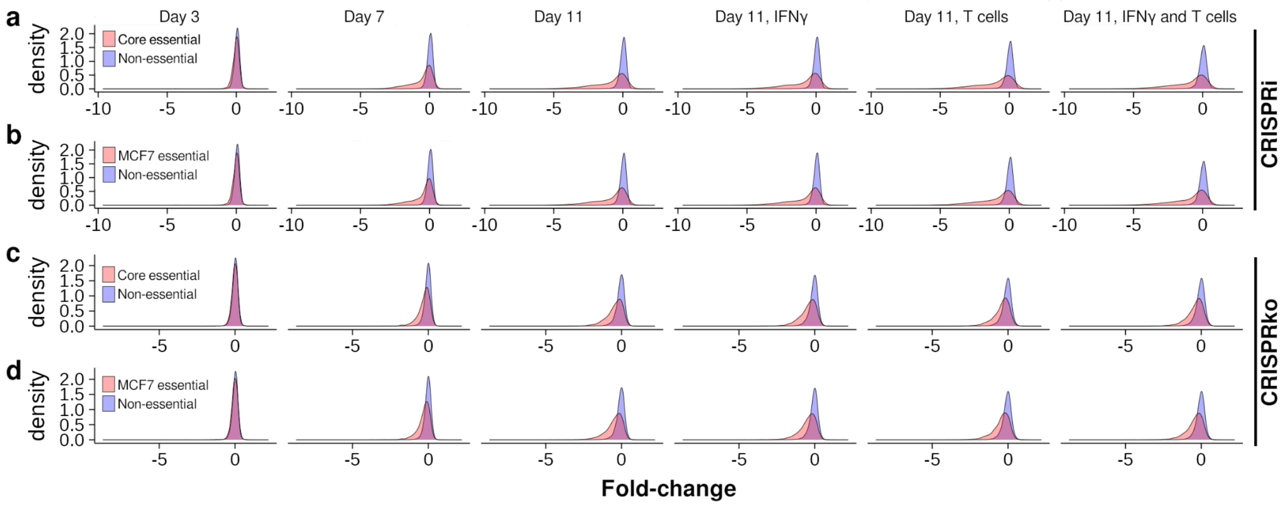

Figure S2. Essential gene depletion. Changes in sgRNA abundances from baseline at timepoints throughout the experimental workflow are represented as fold-changes (mean $\log_2$ count - time 0 count). Genes are categorised as core-essential (**a** and **c**, N = 183), or found to be essential for MCF7 survival in culture from the DepMap project (**b** and **d**, N = 226).

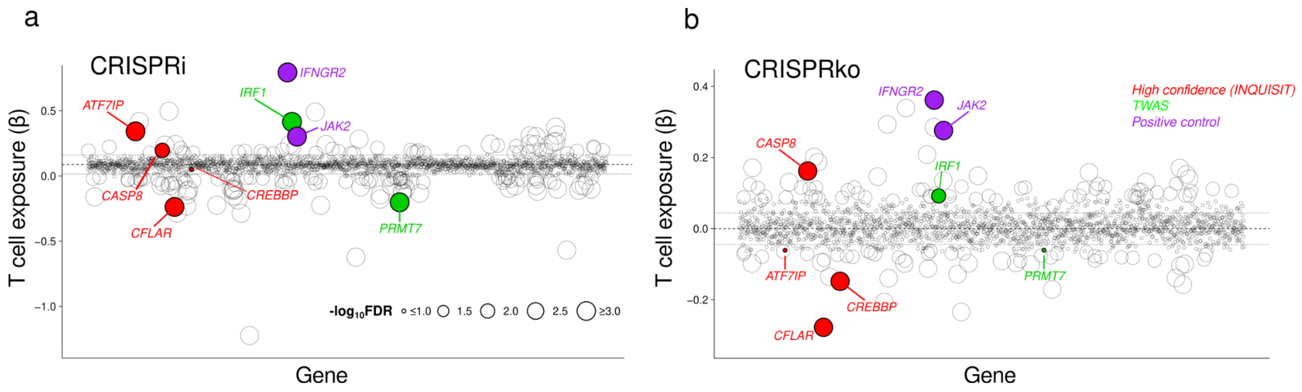

Figure S3. Visualisation of gene-level effects on cell proliferation and T cell killing. Results comparing the β effect of T cell exposure in CRISPR inhibition (**a**) and knockout (**b**) screens. Negative β values are associated with genes that are essential for T cell evasion. Labelled genes were selected for validation. The circle sizes indicate adjusted significance levels and dotted lines show ±1 SD. Label colours indicate the gene prediction method (high confidence INQUISIT red, TWAS green, positive controls purple).

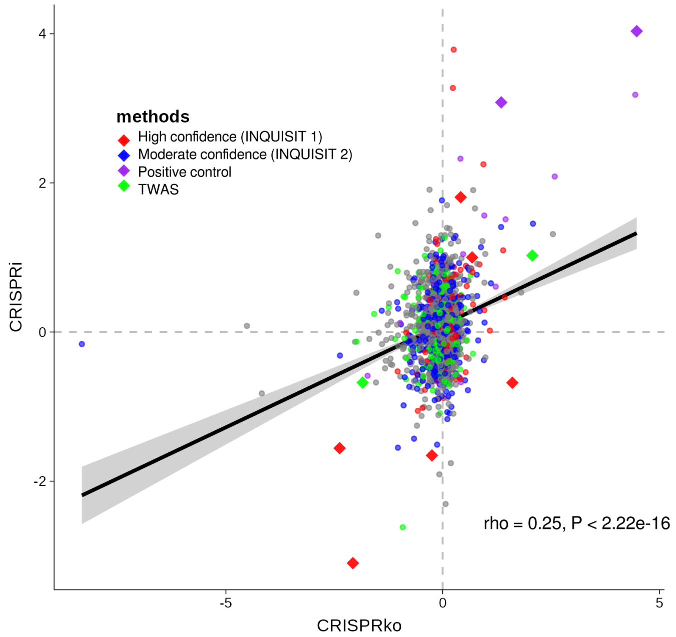

Figure S4. Correlation between effect sizes from CRISPRi and CRISPRko screens. The Pearson coefficient and *P* value for the correlation between libraries is shown, with the genes selected for validation indicated by diamond shaped points. Label colours indicate the gene prediction method (high confidence INQUISIT red, moderate confidence INQUISIT blue, TWAS green, positive controls purple).

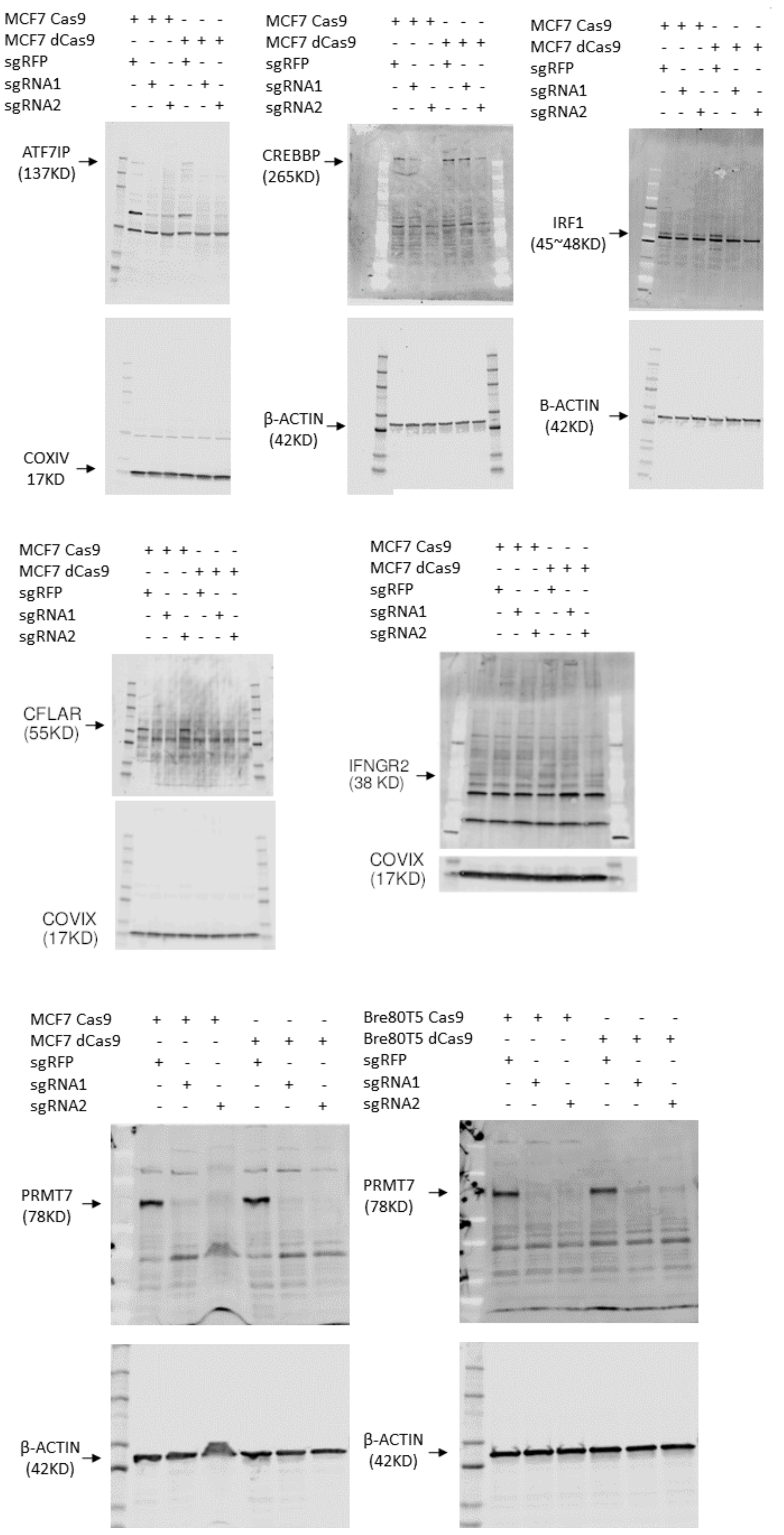

Figure S5. Quantification of target protein levels. Constructs used to infect knockout and inhibition cell lines are labeled above blots. Target proteins and molecular weights are indicated by arrows. Unprocessed scans without any adjustment to image contrast are provided.

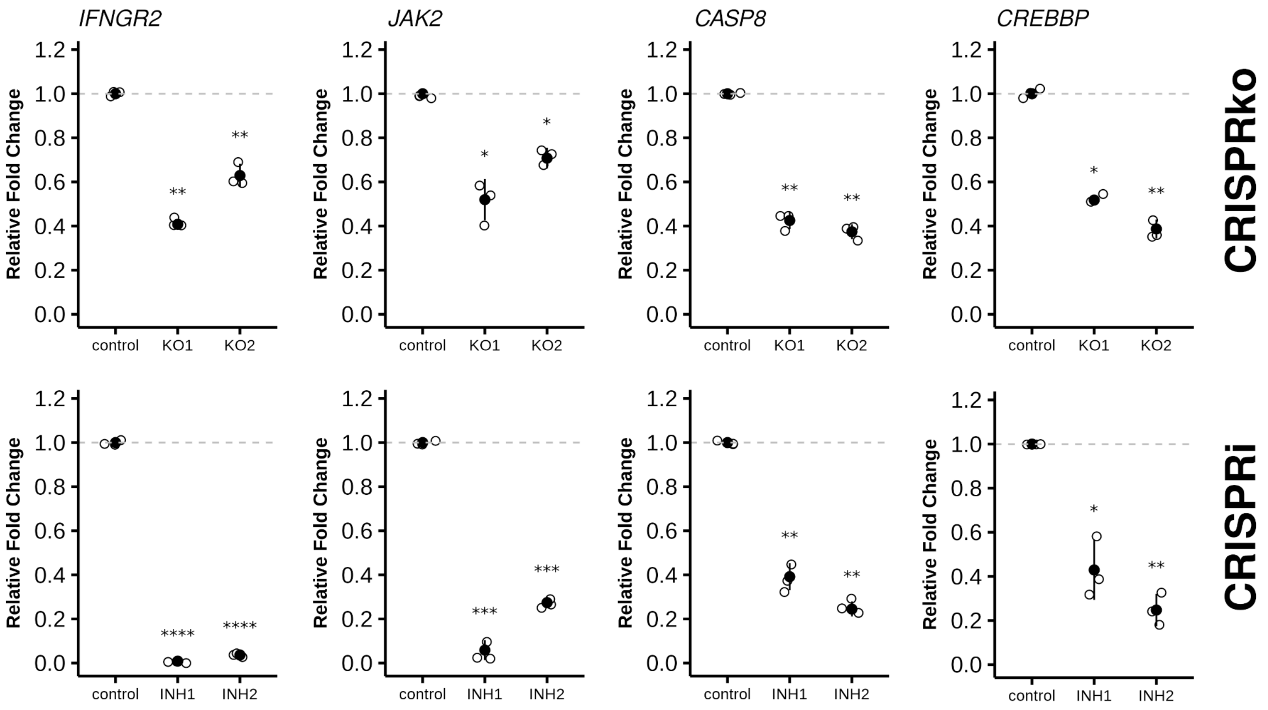

Figure S6. Quantitative PCR measurement of target gene expression. Expression levels in two independent clones were compared to control cells infected with a non-targeting control guide. CRISPRko clones are shown in the top row and CRISPRi clones in the lower row. Filled circles denote the mean relative fold changes ($2^{-\Delta\Delta Ct}$) and whiskers represent standard deviations. Significance was computed with one-sample T tests with $\mu = 1$ using Benjamini-Hochberg correction for multiple testing. All experiments comprised n > 3. (ns = non-significant, $*P < 0.05$, $**P < 0.01$, $***P < 0.001$, $****P < 0.0001$).

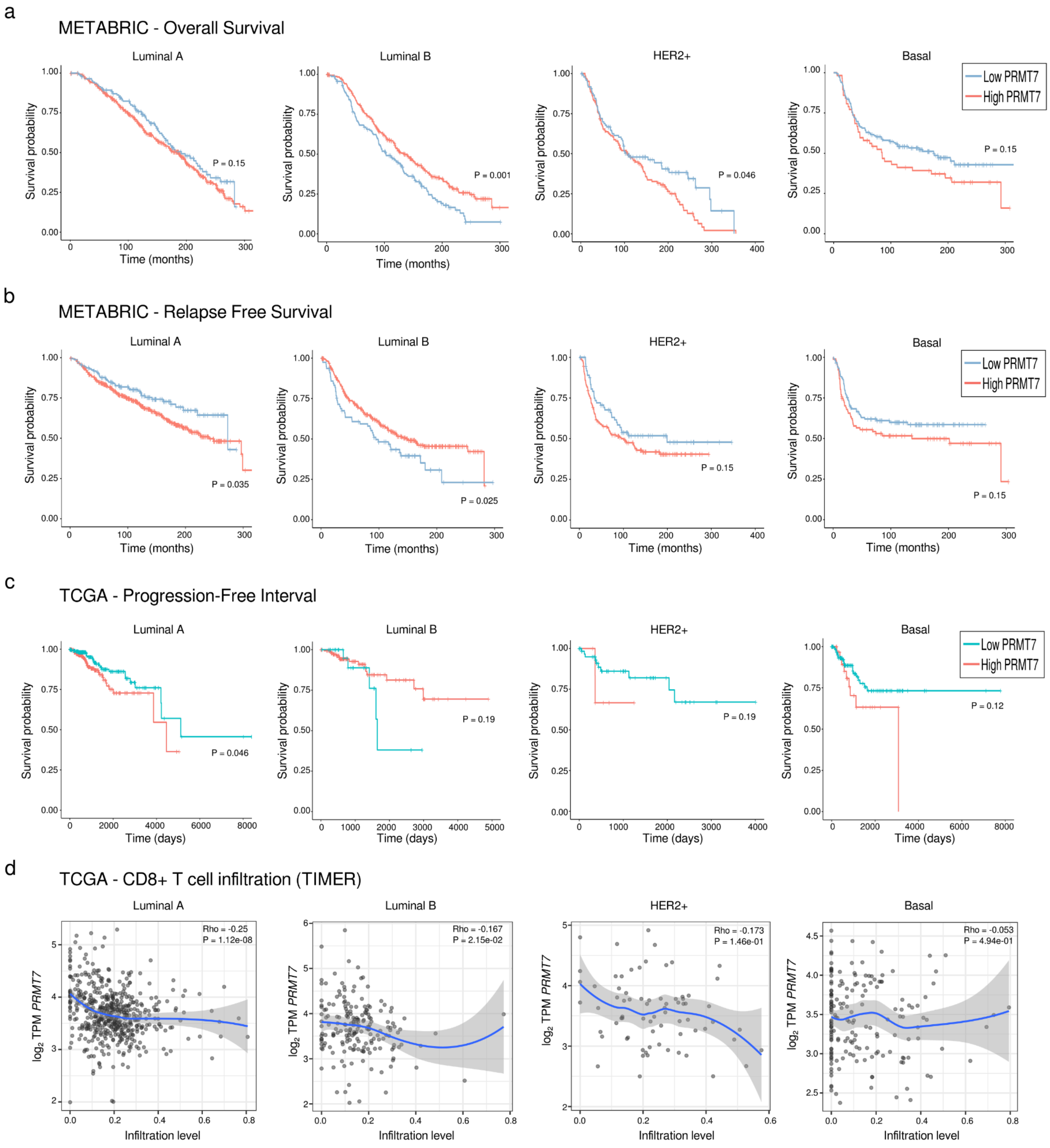

Figure S7. ***PRMT7* expression and survival in breast cancer patients. a**) Examination of *PRMT7* expression and Overall Survival for all subtypes in METABRIC samples. **b**) Relapse-Free Survival in METABRIC. **c**) Association between *PRMT7* and Progression-Free Interval in TCGA tumors. Unadjusted log-rank *P* values are shown. **d**) TIMER analysis of TCGA data showing correlation between *PRMT7* expression and levels of CD8+ T cell infiltration. Spearman correlation coefficients and P values are shown.

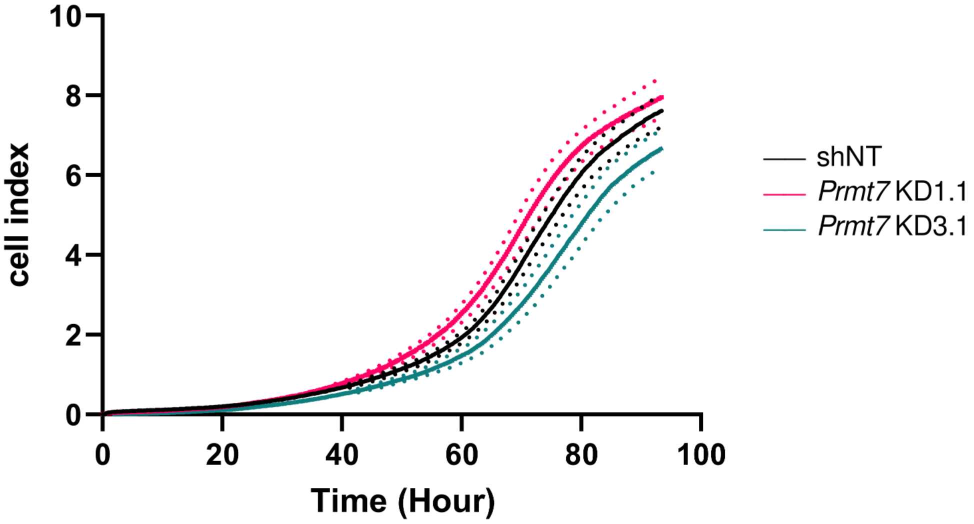

Figure S8. Effect of Prmt7 on *in vitro* proliferation of 4T1 cells. Growth rates of control and shPrmt7-KD clones were measured by xCELLigence RTCA for 96 hours.

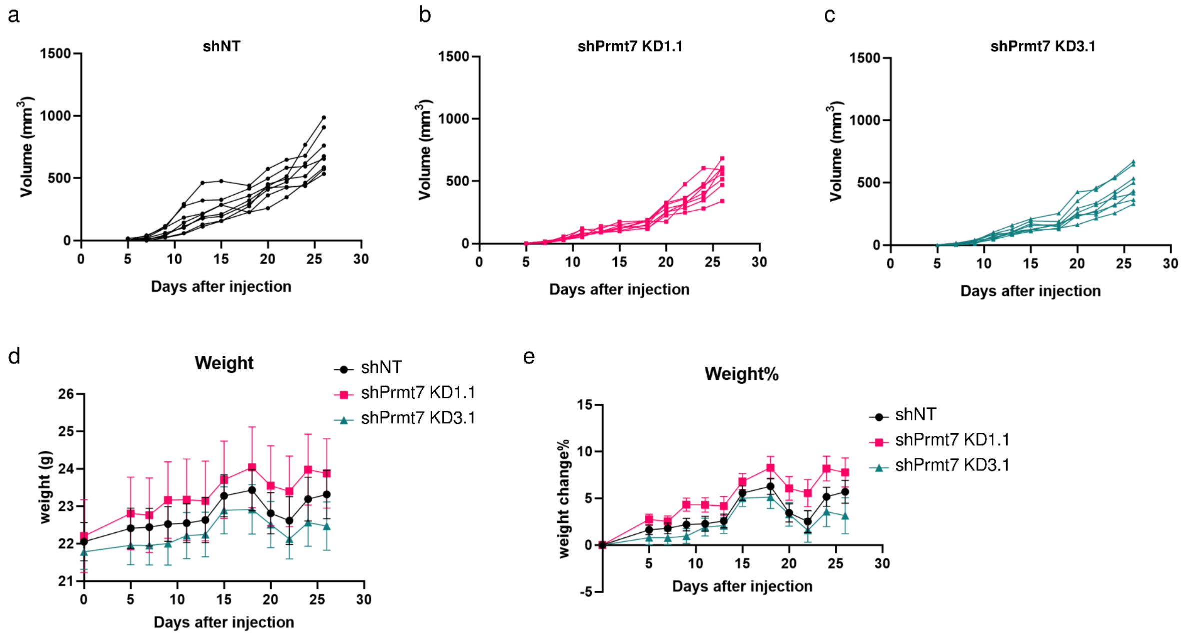

Figure S9. Effect of 4T1 *Prmt7* knockdown on *in vivo* tumor formation in syngeneic models. Tumor measurements for individual BALB/c mice implanted with control (**a**), sh*Prmt7*-KD1.1 (**b**), and sh*Prmt7*-KD3.1 (**c**) cells. Changes in body weight over the experimental course (**d**, **e**).

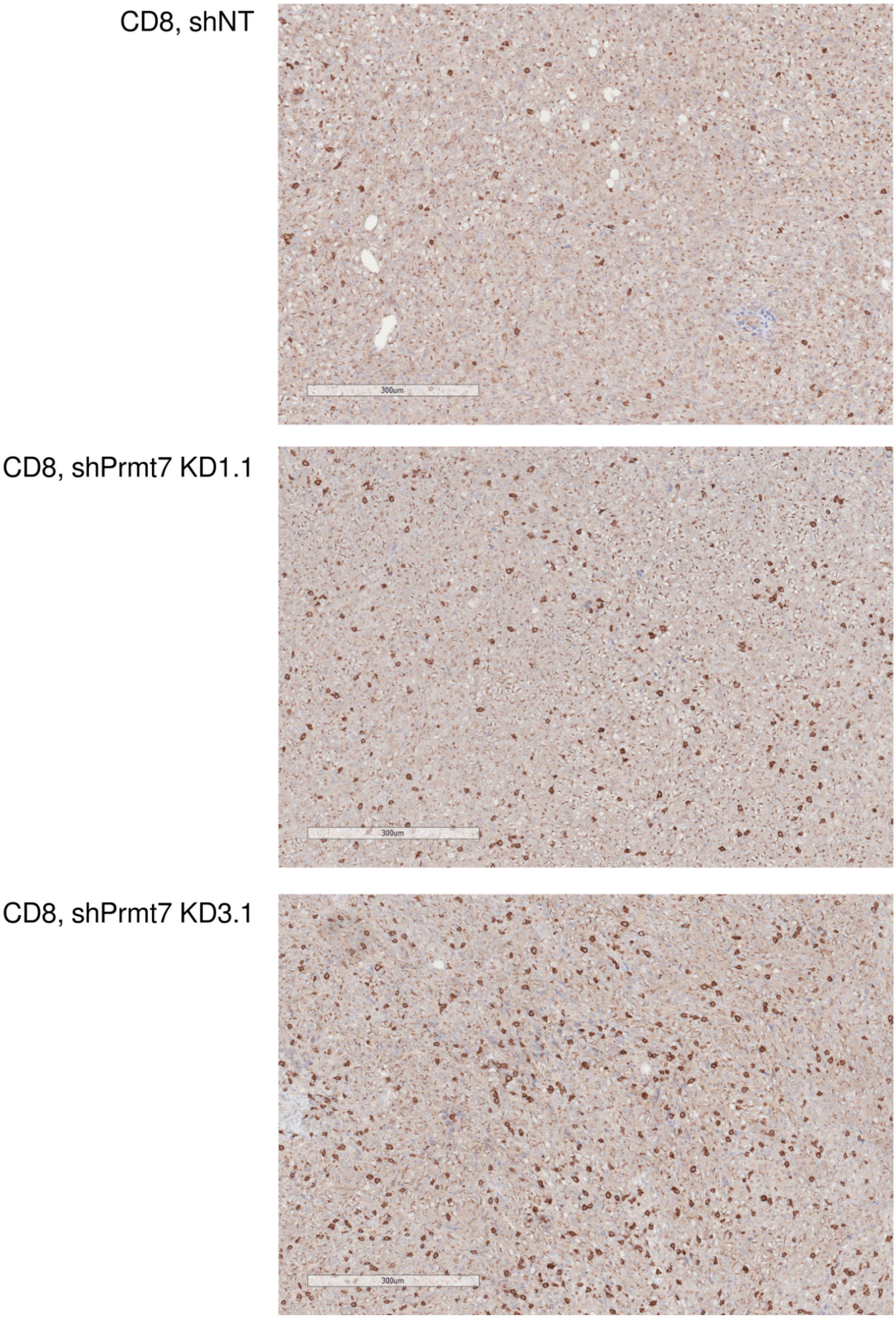

Figure S10. Immunohistochemical staining for CD8+ T cells in endpoint tumors. Representative images of IHC tumour sections stained for CD8 positive T cells in WT, Prmt7 KD1.1, Prmt7 KD3.1 4T1 orthotopic models.

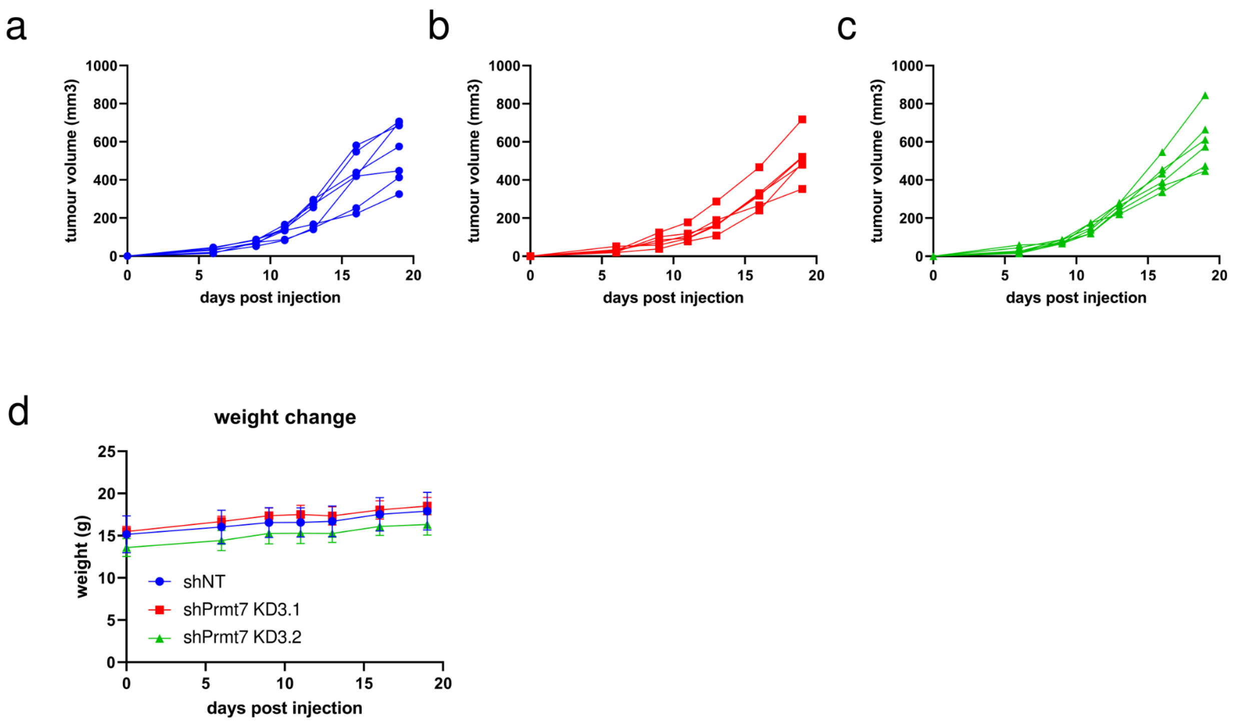

Figure S11. Effect of 4T1 *Prmt7* knockdown on *in vivo* tumor formation in immunocompromised BALB/c nude mice. Tumor measurements for individual mice implanted with control (**a**), sh*Prmt7*-KD3.1 (**b**), and sh*Prmt7*-KD3.2 (**c**) cells. Changes in body weight over the experimental course (**d**).

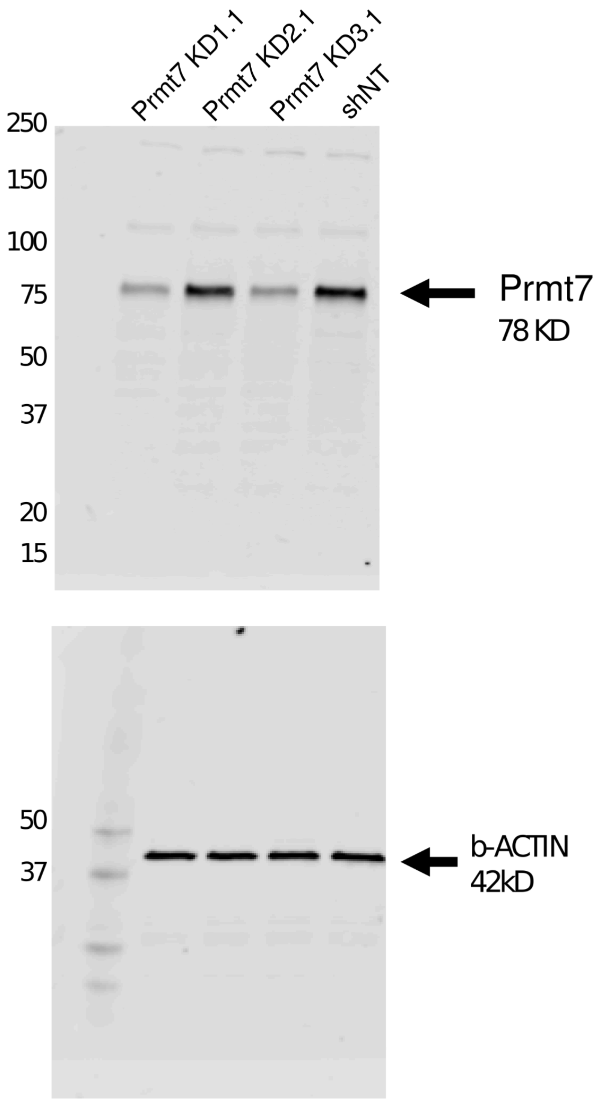

Figure S12. Quantification of Prmt7 target protein levels in 4T1 cells. Target proteins and molecular weights are indicated by arrows. Unprocessed scans without any adjustment to image contrast are provided.

Supplementary Table 1. Primers for single gene validation

Gene	modality	sgRNA Name	sgRNA Sequence
IFNGR2	CRISPRko	IFNGR2_ko1	GACTTGTAAACAGTACACTC
IFNGR2	CRISPRko	IFNGR2_ko2	TTCCGATAGTGTTGAAACCA
IFNGR2	CRISPRi	IFNGR2_inh1	CGTCGCCGCCCAAACCGCCA
IFNGR2	CRISPRi	IFNGR2_inh2	GGTTTGGGCGGCGACGTGAG
JAK2	CRISPRko	JAK2_ko1	CTGCCACTGCAATACCAACG
JAK2	CRISPRko	JAK2_ko2	TCTTCAGGAGAGAATACCAT
JAK2	CRISPRi	JAK2_inh1	CAGCCTGTCAGCGCCGAGGG
JAK2	CRISPRi	JAK2_inh2	GCGCCGAGGGAGGCCGTTAG
CASP8	CRISPRko	CASP8_ko1	GCCTGGACTACATTCCGCAA
CASP8	CRISPRko	CASP8_ko2	CTACCTAAACACTAGAAAGG
CFLAR	CRISPRko	CFLAR_ko1	ATTACCTATAGTCCGAAACA
CFLAR	CRISPRko	CFLAR_ko2	TAATAAATCCAGTTGATCTG
CFLAR	CRISPRi	CFLAR_inh1	GGACTCCCGGAGCTAGGGGT
CFLAR	CRISPRi	CFLAR_inh2	GAGTCCCCACCCCTAGCTCC
IRF1	CRISPRko	IRF1_inh1	CGGGTGGCCTCGGTTCGGCG
IRF1	CRISPRko	IRF1_inh2	GCCCGAGCCCCGCCGAACCG
IRF1	CRISPRi	IRF1_ko1	TTAATTCCAACCAAATCCCG
IRF1	CRISPRi	IRF1_ko2	CACAGTTCCAGGCTACATGC
ATF7IP	CRISPRko	ATF7IP_ko1	AGCAGTATGGAAATTGACCA
ATF7IP	CRISPRko	ATF7IP_ko2	GGGATGGGATCACTAGAGGT
ATF7IP	CRISPRi	ATF7IP_inh1	AAGCTGAGGCGGCAACGTCG
ATF7IP	CRISPRi	ATF7IP_inh2	AAGCGCGCGAAAAGCTGAGG
CREBBP	CRISPRko	CREBBP_ko1	CTTAGCCCACTGATGAACGA
CREBBP	CRISPRko	CREBBP_ko2	TAATTAATCAGGCTTCACAA
CREBBP	CRISPRi	CREBBP_inh1	GGCTTCGAGCGCGATCTACT
CREBBP	CRISPRi	CREBBP_inh2	ATCGCGCTCGAAGCCCCGGT
PRMT7	CRISPRko	PRMT7_ko1	TATCAACAAGCATTCCACCG
PRMT7	CRISPRko	PRMT7_ko2	ACCATACGCAGTAGTCATCG
PRMT7	CRISPRi	PRMT7_inh1	GGTGAGGCGCTGGGTATGCT
PRMT7	CRISPRi	PRMT7_inh2	GGCGAGCGGAGGGTTTCCCG

Supplementary Table 2. Primers for qPCR

Gene	Primer name	Sequence
ACTIN	ACTIN_F	CCAAGGCCAACCGCGAGAAGATGAC
	ACTIN_R	AGGGTACATGGTGGTGCCGCCAGAC
GUSB	GUSB_F	GAAAATATGTGGTTGGAGAGCTCATT
	GUSB_R	CGAGTGAAGATCCCCTTTTTA
PUM1	PUM1_F	AATGCAGGCGCGAGAAAT
	PUM1_R	TTGTGCAGCTGAGGAACTAATGA
JAK2	JAK2_F	TCTGGGGAGTATGTTGCAGAA
	JAK2_R	AGACATGGTTGGGTGGATACC
IFNGR2	IFNGR2_F	CTCCTCAGCACCCGAAGATTC
	IFNGR2_R	GCCGTGAACCATTTACTGTCG
CASP8	CASP8_F	CCAGAGACTCCAGGAAAAGAGA
	CASP8_R	GATAGAGCATGACCCTGTAGGC
CREBBP	CREBBP_F	CAACCCCAAAAGAGCCAAACT
	CREBBP_R	CCTCGTAGAAGCTCCGACAGT

Supplementary Table 3. Genes enriched or depleted upon T cell-mediated tumor cell killing.

Gene	Gene selection criteria	Candidate breast cancer risk gene	Modality	Directio n	Essential	CTL screen hit + candidate breast cancer risk gene
ATF7IP	INQ_1	TRUE	CRISPRi	sensitiser	FALSE	TRUE
CASP8	INQ_1	TRUE	CRISPRko	sensitiser	FALSE	TRUE
CDKAL1	INQ_1	TRUE	CRISPRko	resistor	FALSE	TRUE
CFLAR	INQ_1	TRUE	CRISPRko, CRISPRi	resistor	FALSE	TRUE
CREBBP	INQ_1	TRUE	CRISPRko	resistor	FALSE	TRUE
HSPA4	INQ_1	TRUE	CRISPRi	resistor	FALSE	TRUE
NF1	INQ_1	TRUE	CRISPRko	resistor	FALSE	TRUE
SMG9	INQ_1	TRUE	CRISPRi	sensitiser	FALSE	TRUE
SOX13	INQ_1	TRUE	CRISPRi	sensitiser	FALSE	TRUE
TCF7L2	INQ_1	TRUE	CRISPRi	sensitiser	FALSE	TRUE
TGFBR2	INQ_1	TRUE	CRISPRi	sensitiser	FALSE	TRUE
AC068831.6	INQ_2	TRUE	CRISPRko	resistor	FALSE	TRUE
AZIN1	INQ_2	TRUE	CRISPRko, CRISPRi	sensitiser	FALSE	TRUE
CLK1	INQ_2	TRUE	CRISPRko	resistor	FALSE	TRUE
IL6ST	INQ_2	TRUE	CRISPRi	sensitiser	FALSE	TRUE
LIN54	INQ_2	TRUE	CRISPRko	resistor	FALSE	TRUE
MAP3K11	INQ_2	TRUE	CRISPRi	sensitiser	FALSE	TRUE
ORC2	INQ_2	TRUE	CRISPRko	resistor	FALSE	TRUE
RBM8A	INQ_2	TRUE	CRISPRi	resistor	FALSE	TRUE
RP11-14D22.2	INQ_2	TRUE	CRISPRko	sensitiser	FALSE	TRUE
SEC22B	INQ_2	TRUE	CRISPRi	resistor	FALSE	TRUE
SOX4	INQ_2	TRUE	CRISPRko, CRISPRi	resistor	FALSE	TRUE
SP3	INQ_2	TRUE	CRISPRi	sensitiser	FALSE	TRUE
SRSF9	INQ_2	TRUE	CRISPRko	resistor	FALSE	TRUE
TFAP4	INQ_2	TRUE	CRISPRi	sensitiser	FALSE	TRUE
AC007283.5	TWAS	TRUE	CRISPRi	resistor	FALSE	TRUE
COX11	TWAS	TRUE	CRISPRi	resistor	FALSE	TRUE

DDA1	TWAS	TRUE	CRISPRi	resistor	FALSE	TRUE
IRF1	TWAS	TRUE	CRISPRi	sensitiser	FALSE	TRUE
LMO4	TWAS	TRUE	CRISPRko	resistor	FALSE	TRUE
MAEA	TWAS	TRUE	CRISPRko	resistor	FALSE	TRUE
NPAT	TWAS	TRUE	CRISPRi	resistor	FALSE	TRUE
PRMT7	TWAS	TRUE	CRISPRi	resistor	FALSE	TRUE
CCND1	INQ_1	TRUE	CRISPRko, CRISPRi	sensitiser	TRUE	FALSE
ESR1	INQ_1	TRUE	CRISPRko	sensitiser	TRUE	FALSE
EWSR1	INQ_1	TRUE	CRISPRko, CRISPRi	resistor	TRUE	FALSE
GATA3	INQ_1	TRUE	CRISPRko	sensitiser	TRUE	FALSE
CHMP6	INQ_2	TRUE	CRISPRi	resistor	TRUE	FALSE
FDPS	INQ_2	TRUE	CRISPRi	resistor	TRUE	FALSE
UPF1	INQ_2	TRUE	CRISPRi	resistor	TRUE	FALSE
POLR2L	TWAS	TRUE	CRISPRi	resistor	TRUE	FALSE
AC092069.1	Background	FALSE	CRISPRko	sensitiser	FALSE	FALSE
CCDC103	Background	FALSE	CRISPRi	resistor	FALSE	FALSE
GAD1	Background	FALSE	CRISPRko	resistor	FALSE	FALSE
METAP2	Background	FALSE	CRISPRko	sensitiser	FALSE	FALSE
PLEKHA1	Background	FALSE	CRISPRko	resistor	FALSE	FALSE
RP3-443C4.2	Background	FALSE	CRISPRi	sensitiser	FALSE	FALSE
SUDS3	Background	FALSE	CRISPRko	resistor	FALSE	FALSE
TOMM70	Background	FALSE	CRISPRi	resistor	FALSE	FALSE
LSM6	Core essential	FALSE	CRISPRi	resistor	FALSE	FALSE
SMC3	Core essential	FALSE	CRISPRi	sensitiser	FALSE	FALSE
U2AF2	Core essential	FALSE	CRISPRi	resistor	FALSE	FALSE
CDC5L	Core essential	FALSE	CRISPRi	resistor	TRUE	FALSE
CHD4	Core essential	FALSE	CRISPRi	sensitiser	TRUE	FALSE
CHMP2A	Core essential	FALSE	CRISPRi	resistor	TRUE	FALSE

COPS6	Core essential	FALSE	CRISPRi	resistor	TRUE	FALSE
COPS8	Core essential	FALSE	CRISPRi	resistor	TRUE	FALSE
COPZ1	Core essential	FALSE	CRISPRi	resistor	TRUE	FALSE
EEF2	Core essential	FALSE	CRISPRko	sensitiser	TRUE	FALSE
EFTUD2	Core essential	FALSE	CRISPRi	resistor	TRUE	FALSE
EIF3A	Core essential	FALSE	CRISPRi	resistor	TRUE	FALSE
EIF3D	Core essential	FALSE	CRISPRi	resistor	TRUE	FALSE
EIF3F	Core essential	FALSE	CRISPRi	resistor	TRUE	FALSE
EIF3G	Core essential	FALSE	CRISPRi	resistor	TRUE	FALSE
HNRNPC	Core essential	FALSE	CRISPRko	resistor	TRUE	FALSE
NEDD8	Core essential	FALSE	CRISPRi	resistor	TRUE	FALSE
PHB	Core essential	FALSE	CRISPRi	resistor	TRUE	FALSE
PHB2	Core essential	FALSE	CRISPRi	resistor	TRUE	FALSE
PSMA1	Core essential	FALSE	CRISPRi	resistor	TRUE	FALSE
PSMA6	Core essential	FALSE	CRISPRi	resistor	TRUE	FALSE
PSMD1	Core essential	FALSE	CRISPRi	resistor	TRUE	FALSE
RPS19	Core essential	FALSE	CRISPRko	sensitiser	TRUE	FALSE
RRM1	Core essential	FALSE	CRISPRi	resistor	TRUE	FALSE
SRSF1	Core essential	FALSE	CRISPRko	sensitiser	TRUE	FALSE
SUPT6H	Core essential	FALSE	CRISPRko	sensitiser	TRUE	FALSE
VCP	Core essential	FALSE	CRISPRi	resistor	TRUE	FALSE

XRCC6	Core essential	FALSE	CRISPRi	resistor	TRUE	FALSE
TFAP2C	Non-background	FALSE	CRISPRi	sensitiser	TRUE	FALSE
THOC5	Non-background	FALSE	CRISPRi	resistor	TRUE	FALSE
CD58	Positive control	FALSE	CRISPRko, CRISPRi	sensitiser	FALSE	FALSE
ERAP1	Positive control	FALSE	CRISPRi	resistor	FALSE	FALSE
HLA-A	Positive control	FALSE	CRISPRko	sensitiser	FALSE	FALSE
IFNGR1	Positive control	FALSE	CRISPRko, CRISPRi	sensitiser	FALSE	FALSE
IFNGR2	Positive control	FALSE	CRISPRko, CRISPRi	sensitiser	FALSE	FALSE
JAK1	Positive control	FALSE	CRISPRko, CRISPRi	sensitiser	FALSE	FALSE
JAK2	Positive control	FALSE	CRISPRko, CRISPRi	sensitiser	FALSE	FALSE
NLRC5	Positive control	FALSE	CRISPRi	sensitiser	FALSE	FALSE
TAP1	Positive control	FALSE	CRISPRko, CRISPRi	sensitiser	FALSE	FALSE
